# Limited genomic consequences of hybridization between two African clawed frogs, *Xenopus gilli* and *X*. *laevis* (Anura: Pipidae)

**DOI:** 10.1038/s41598-017-01104-9

**Published:** 2017-04-24

**Authors:** Benjamin L. S. Furman, Caroline M. S. Cauret, Graham A. Colby, G. John Measey, Ben J. Evans

**Affiliations:** 1 Biology Department, Life Sciences Building room 328, McMaster University, 1280 Main Street West, Hamilton, ON L8S 4K1 Canada; 2 Centre for Invasion Biology, Department of Botany and Zoology, Stellenbosch University, Private Bag X1, Matieland, 7602 Stellenbosch South Africa

## Abstract

The Cape platanna, *Xenopus gilli*, an endangered frog, hybridizes with the African clawed frog, *X*. *laevis*, in South Africa. Estimates of the extent of gene flow between these species range from pervasive to rare. Efforts have been made in the last 30 years to minimize hybridization between these two species in the west population of *X*. *gilli*, but not the east populations. To further explore the impact of hybridization and the efforts to minimize it, we examined molecular variation in one mitochondrial and 13 nuclear genes in genetic samples collected recently (2013) and also over two decades ago (1994). Despite the presence of *F*_1_ hybrids, none of the genomic regions we surveyed had evidence of gene flow between these species, indicating a lack of extensive introgression. Additionally we found no significant effect of sampling time on genetic diversity of populations of each species. Thus, we speculate that *F*_1_ hybrids have low fitness and are not backcrossing with the parental species to an appreciable degree. Within *X*. *gilli*, evidence for gene flow was recovered between eastern and western populations, a finding that has implications for conservation management of this species and its threatened habitat.

## Introduction

Gene flow (introgression) between species may facilitate adaptive evolution through the exchange of beneficial genetic variation. This expedites the colonization of specialized ecological niches^1–3^, and affects future adaptive potential by increasing genetic and phenotypic variation^2, 4–7^. However, gene flow between species also poses risks by eroding species boundaries^8^, disrupting adaptively evolved complexes of alleles^9, 10^, promoting the exchange of genetic variation associated with disease^11^, influencing pathogen emergence^12^, and facilitating species invasion^13, 14^. As such, hybridization has important implications for biodiversity conservation.

### Hybridization in African clawed frogs

Hybridization features prominently in the evolutionary history of African clawed frogs (genus *Xenopus*); 28 of 29 species are polyploid, and all of these are probably allopolyploid^15, 16^. When backcrossed in the laboratory, there is variation among *F*_1_ *X*. *gilli*-*laevis* hybrid females with respect to whether or not their progeny are polyploid^17^. Laboratory studies indicate that in some crosses (*X*. *gilli*-*X*. *laevis* and *X*. *laevis*-*X*. *muelleri*) *F*_1_ hybrid males are sterile, but female *F*_1_ hybrids are fertile^17, 18^. *F*_1_ *X*. *gilli*-*X*. *laevis* hybrid females are capable of backcrossing with either parental species, and both sexes of the *F*_2_ backcross generation can be fertile^17^. Thus there exists the possibility that gene flow among *Xenopus* species could occur in nature. At least three *Xenopus* hybrid zones are thought to exist^19–21^, and hybrids in each of these zones may have the same ploidy level as the parental species (pseudotetraploid; ref. 22).

### The *X*. *gilli*/*X*. *laevis* hybrid zone

**Figure 1:**
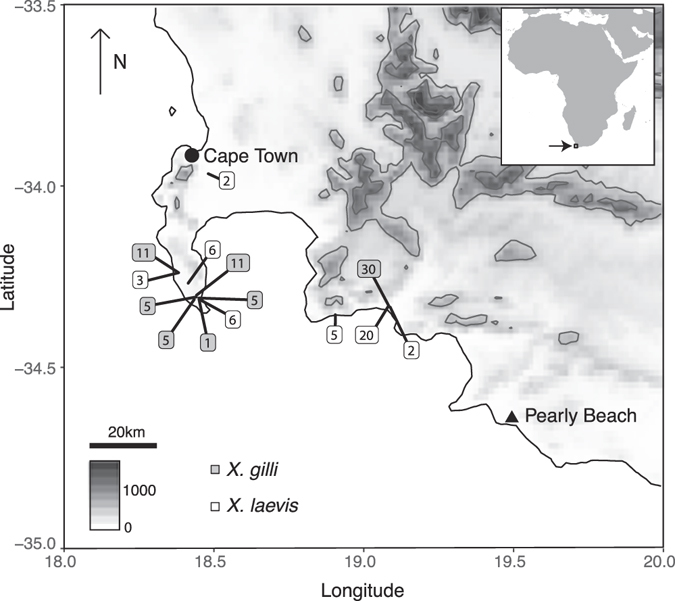
Sampling locations. For each species, numbers indicate the sum of number of individuals from each locality sampled in 1994 and 2013. An inset indicates the study area in southern Africa and altitude in meters is indicate on the scale. The map was made using the R package Marmap^81^ using topographic data from the National Oceanic and Atmospheric Administration, USA.

Classified by the IUCN as Endangered^23^, *X. gilli* ^24^ occurs in southwestern Western Cape Provence, South Africa^25–28^. *Xenopus gilli* is a found in seasonal ponds in lowland coastal fynbos habitat, a component of the Cape Floristic Region, which is a biodiversity hotspot^29^ with an extreme level of plant endemism^30^. These ponds have high concentrations of humic compounds derived from the surrounding fynbos vegetation, and a characteristic dark color and low pH^25, 31, 32^. The range of *X*. *gilli* is disjunct and includes the Cape of Good Hope section of Table Mountain National Park (CoGH), habitat near the town of Kleinmond, and habitat near the town of Pearly Beach (refs 25–28; Fig. 1). These three localities are interrupted by unsuitable, highly modified habitat that may impede contemporary gene-flow^26^. As with many amphibians^33^, habitat degradation is a major threat to *X*. *gilli* ^25, 26^.

In contrast, *X*. *laevis* ^34^, is found throughout southern Africa, in both natural and disturbed areas of South Africa and Malawi^35, 36^. *Xenopus laevis* is syntopic throughout the range of *X*. *gilli* ^25–28^ and can tolerate a broad spectrum of environmental challenges including extremes of desiccation, salinity, anoxia, and temperature^37^. Picker *et al.* ^32^proposed that there may be an ecological basis for speciation of *X*. *laevis* and *X*. *gilli* centered on higher tolerance of *X*. *gilli* embryos to low pH, allowing for habitat specialization.

Several aspects of external morphology readily distinguish these species, including smaller size of *X*. *gilli*, the presence of longitudinal dorsal mottling that does not connect over the midline in *X*. *gilli* only (for example, see Fig. 1 of ref. 19), and orange and black vermiculation on the venter of *X*. *gilli*. *F*_1_ hybrids between *X*. *gilli* and *X*. *laevis* are readily identified based on individuals that are morphologically intermediate with respect to size and coloration, and this identification has been confirmed by molecular tests^19, 28, 38, 39^. The reported abundance of *F*_1_ hybrids varies from relatively common^26, 38, 39^, to rare^27, 28^. Morphological variation of some individuals has been previously interpreted as being derived from backcrosses of *F*_1_ hybrids with each parental species^39^.

The western extent of the *X*. *gilli* distribution occurs within the CoGH^25, 26^. Following reports of hybrids and expansion of *X*. *laevis* populations, steps were taken in the mid-1980s to minimize co-occurence of these two species within the CoGH which included removal of *X*. *laevis* from *X*. *gilli* ponds, translocation of *X*. *gilli* to new sites^40^, and construction of a wall around a known *X*. *gilli* pond^25, 41^. The hope was to minimize hybridization and resource competition, for example, if larger *X*. *laevis* individuals are able to outcompete *X*. *gilli* for food^39, 42^. With some interruptions, these efforts have continued for the last 30 years in the CoGH. Similar efforts have not been made for eastern populations of *X*. *gilli* which are located on private property, and in some ponds in these areas where *X*. *gilli* had been found in the past, now only *X*. *laevis* are found^26, 41^.

To further investigate the effect of hybridization on gene flow between *X*. *gilli* and *X*. *laevis*, we examined DNA sequence variation in these species from one mitochondrial DNA (mtDNA) marker and 13 nuclear DNA (nDNA) markers. Genetic samples were collected from within managed (west) and unmanaged (east) portions of the range of *X*. *gilli*. Samples were analyzed from both locations that were collected shortly after management began, and then in the same areas again 20 years later. We expected that if introgression was occurring in the populations during this time period, it would be more pronounced in the east population. If efforts to minimize hybridization in the west were successful, we expected more evidence of gene flow in the samples collected soon after management began as compared to more recently. However, in both localities and both sampling times, we found no evidence of shared mitochondrial haplotypes or nuclear alleles between these species, suggesting that the *F*_1_ hybrids have low fitness and are not backcrossing with the parental species to an appreciable degree, despite potential fertility of *F*_1_ females^17^. Within *X*. *gilli*, we recovered evidence of gene flow between east and west populations, and found genetic diversity to be higher in the unprotected eastern population. These findings have implications for management and conservation of this endangered habitat specialist.

## Materials and Methods

Genetic samples analyzed in this study were collected either in 1994 or in 2013. Some of the samples from the earlier collection were also analyzed in two earlier studies^27, 28^. The 2013 collection included *X*. *gilli* and *X*. *laevis* individuals from the same or geographically close (within 5 km) sites as the 1994 collection, and both sampling efforts used funnel traps. Animal sampling protocols approved by the Institutional Animal Care and Use Committee at Columbia University and work was performed in accordance with all relevant guidelines and regulations for animal experimentation, in accordance with laws for studying wildlife in South Africa and with appropriate collection permits from the Chief Directorate of Nature Conservation and Museums, and was approved by the Animal Ethics Committee at the University of Cape Town and the Stellenbosch University Research Ethics Committee: Animal Care and Use. Samples were obtained east and west of False Bay for both species and for both time periods (Fig. 1). We assigned individuals to species (*X*. *gilli* or *X*. *laevis*) based on dorsal and ventral patterning, shape of head, and overall size^43, 44^. Because this study aimed to explore genetic effects of backcrossed hybrids, for both time points, we intentionally excluded individuals whose intermediate morphology (and genetic analysis in the case of the 1994 individual^28^) indicated that they were *F*_1_ hybrids (1 individual from 1994 and 9 from 2013).

DNA was extracted from tissue samples using Qiagen DNEasy tissue extraction kits (Qiagen, Inc), following the manufacturer’s protocol, or a phenol-chloroform protocol. A fragment of the mtDNA genome was amplified and sequenced for 36 and 33 *X*. *gilli* and *X*. *laevis* individuals, respectively, using primers from ref. 45 that target a portion of the 16S ribosomal RNA gene (*16S*). Exons of 13 nDNA genes ranging from 333–770 bp in length were sequenced for 20–41 *X*. *gilli* and 11–31 *X*. *laevis* individuals using paralog specific primers (primers are from ref. 46). These exons came from the genes B-Cell CLL/Lymphoma 9 (*BCL9*), BTB domain containing 6 (*BTBD6*), Chromosome 7 Open Reading Frame 25 (*C7orf25*), Fem-1 Homolog C (*FEM1C*), Microtubule Associated Serine/Threonine Kinase Like (*MASTL*), Mannosyl-oligosaccharide glucosidase (*MOGS*-*1*), Nuclear Factor, Interleukin 3 Regulated (*NFIL*-*3*), protocadherin 1 (*PCDH1*), phosphatidylinositol glycan anchor biosynthesis class O (*PIGO*), protein arginine methyltransferase 6 (*PRMT6*), Ras association domain family member 10 (*RASSF10*), SURP and G-patch domain containing 2 (*SUGP2*), and zinc finger BED-type containing 4 (*ZBED4*). A table of sample IDs and which loci were amplified for which samples is available in the Appendix. In the phylogenetic analysis of individual genes (discussed below), we used as an outgroup a sequence from *X. tropicalis* from the genome assembly version 9.0 on Xenbase^47^. When possible, we also included orthologous and homeologous sequences from *X*. *laevis* from the genome assembly version 9.1 on Xenbase^47^, which was identified using BLAST^48^; this was not possible when a homeologous sequence was not identified, which could be due to gene loss or missing data in the genome sequence. Sequence data were aligned using MAFFT^49^ and corrected by eye. Coding frame was estimated using the ‘minimize stop codons’ option in Mesquite v.3.04^50^, and alignments were trimmed to begin at the first position and end at the third position of the reading frame.

We calculated the phase of nDNA alleles (i.e. haplotypes) using the ‘best guess’ option of PHASE^51, 52^ with default parameters. Each individual’s allelic sequences for each locus were used in subsequent population genetic, clustering, and gene tree analyses. Thus, for each nuclear locus, an individual frog was represented by two sequences, each corresponding to one allele.

### Gene trees

Gene trees were estimated for each phased nDNA exon and the mtDNA alignment using BEAST v1.8.3^53^. Substitution models were selected based on the Akaike Information Criterion using MRMODELTEST v.2^54^, and xml files were prepared for BEAST using BEAUTI (part of the BEAST package). For each nDNA locus, we ran two Markov chain Monte Carlo runs for 25 million generations. For the mtDNA, the model selected by MRMODELTEST2 (GTR+Γ) failed to converge on stable parameter estimates, and we therefore instead used the simpler HKY+Γ model, and ran two chains for 50 million generations. For each analysis, convergence of parameter estimates on the posterior distribution was assessed using TRACER v.1.5^55^ based on an effective sample size (ESS) value >200 and inspection of the trace of parameter estimates against the MCMC generation number. Based on this, for all phylogenetic analyses the first 25% of the posterior distribution was discarded as burn-in. Then, using TREEANNOTATOR, we produced consensus trees from the post-burn-in posterior distribution of trees.

### Species tree

We also estimated a species tree (with the nuclear sequences used in the STRUCTURE analysis, see below) using the multi-species coalescent model of *BEAST^56^. We trimmed the dataset to include only  nDNA genes with all populations sampled (see Genetic clusters section for details) and included only individuals sampled for all genes. All *X*. *laevis* individuals were considered to be the same species (17 individuals), and we separated the east and west *X*. *gilli* populations into separate species (10 and 11 individuals, respectively), and *X*. *tropicalis* was considered its own species. We set a simple HKY+*Γ* model joined for all data partitions (so that convergence of parameter estimates could be reached), assumed a strict molecular clock joined for all data partitions, and allowed the underlying gene tree structure to vary across data partitions. We ran the 8 chains for 170 million generations and removed 50 million generations as burn-in.

### Genetic clusters

We used STRUCTURE v.2.3.4^57^ to estimate individual assignment probabilities to genetic clusters using best-guess phased nDNA alleles on a subset of individuals. Three loci lacked data from the east *X*. *gilli* 1994 population (exons of the genes *MOGS*-*1*, *PCDH1* that also lacked data from this exon for *X*. *laevis* east 1994 samples, and *PIGO*), so we excluded them from STRUCTURE analysis. We also excluded individuals with >50% missing data for the remaining 10 loci. This resulted in a dataset of 13, 8, 11, and 6 *X*. *gilli* individuals from the following localities and years respectively: east 1994, east 2013, west 1994, and west 2013, where east and west refer to the sampling locations relative to False Bay. This analysis also included 9, 12, 6, 4 *X*. *laevis* individuals from east 1994, east 2013, west 1994, and west 2013 respectively. We used the admixture model of STRUCTURE and assumed no correlation between alleles at different loci. We ran the Markov chain Monte Carlo for 20 million generations, following a two million generation burn-in. We tested a number of clusters (*K*) ranging from 1–8, with 5 replicate analyses for each setting of *K*. To correct for label switching and to average assignment probabilities across runs, we used CLUMPP v.1.1.2^58^. We first computed the *D* statistic, following recommendations in the CLUMPP manual^58^, to decide on the particular algorithm to employ for maximizing similarity across runs. We then used the *ad hoc* method of ref. 57 and the method described by ref. 59 to evaluate the most likely number of genetic clusters (*K*).

### Evolutionary models

**Figure 2:**
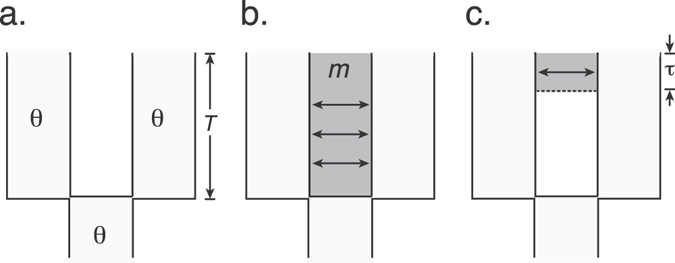
Evolutionary models considered for *X*. *gilli* sequence data from east and west populations included (**a**) population division without subsequent gene flow, (**b**) separation followed by ongoing gene flow, and (**c**) separation followed by secondary contact after a period of no gene flow. Model parameters include the population polymorphism parameter *θ*, which is assumed to be constant in the ancestral and both descendant populations, the time of speciation *T*, the amount of migration *m*, and the time of secondary contact *τ*.

As discussed below, our analyses did not detect mitochondrial DNA haplotypes or nuclear alleles that were shared between *X*. *laevis* and *X*. *gilli* but did detect shared haplotypes and alleles between populations of *X*. *gilli* and between populations of *X*. *laevis*. Because *X*. *gilli* is of conservation concern, we evaluated the fit of data from this species to three evolutionary models (Fig. 2). In the first (isolation) model, divergence of two *X*. *gilli* populations was followed by no migration between each population. Under the isolation model, all shared alleles between these populations would be due to incomplete lineage sorting (ILS). In the second (ongoing migration) model, *X*. *gilli* population divergence was followed by ongoing symmetrical migration between the populations. Under the ongoing migration model, shared alleles would be due to ILS or migration, and some of the alleles shared due to migration could have been exchanged millions of years ago. In the third (secondary contact) model, *X*. *gilli* population divergence was followed by a period of no migration and then by a period during which symmetrical migration occurred between east and west populations. Under the secondary contact model, shared alleles would again be due to ILS or migration, but alleles shared due to migration could only have been exchanged recently.

All models include a parameter *T*, which is the time of separation between the *X*. *gilli* populations and a parameter *θ*, which is the population polymorphism parameter of the ancestral and both descendant populations. The second and third models have an additional parameter *m*, which is the number of individuals in each population that are replaced per generation by individuals from the other population (east vs west), divided by the product of four times the effective population size of each population. The third model includes another parameter *τ*, which is the proportion of *T* going back in time from the present that secondary contact began. Thus, the ongoing migration and isolation models are special cases of the secondary contact model in which *τ* = 1, or *τ* = 1 and *m* = 0, respectively. We note that several assumptions of these models are undoubtedly violated (e.g., constancy of population size over time, equivalent population size of both descendant and the ancestral populations) but we made them nonetheless so we could complete the simulations (see below) within a reasonable amount of time, and because of the relatively small size of the dataset.

The approximate likelihood of combinations of values for these parameters was estimated using rejection sampling^60^. In this approach, the likelihood is approximated by the natural logarithm of the number of simulations for which the sum of four summary statistics from a simulation (discussed next) were within ±ε % of the sum of the observed four summary statistics from actual sequence data, divided by the number of simulations, where *ε* = 25. The value of *ε* determined how close the simulations must match the observed data in order to contribute to the likelihood, and was selected based on a compromise between the computational efficiency of the likelihood estimation and the accuracy of the estimate^60^. For the ongoing migration model and the secondary contact model, 40,000 simulations were performed for each combination of parameter values we considered. For the isolation model, no simulations had summary statistics within ±ε % of the observed; thus, 1,000,000 simulations were performed in order to achieve an upper bound for the likelihood estimate. The likelihood of the data over all combinations of the following parameter value intervals were estimated: *T*: every 1,000,000 generations in the interval of 0–20,000,000 generations; *θ*: every 0.001 units in the interval of 0.001–0.01 and every 0.01 units in the interval of 0.01–0.1; *τ*: every 10% in the interval of 10–100%; *m* every 0.1 units in the interval of 0–1 and every integer in the interval of 1–10.

We used the sum across loci of four summary statistics described by ref. 61 for these likelihood calculations, and simulations were performed using the program mimarsim^62^. These four summary statistics include the number of sites with a derived polymorphism (i) in the west population of *X*. *gilli* only, (ii) in the east population of *X*. *gilli* only, (iii) shared between the west and east populations of *X*. *gilli*, or (iv) fixed in either the west or in the east population of *X*. *gilli*. The simulations used a fixed value for the mutation rate equal to 2.69*e* ^−9^ substitutions per site per generation, which was estimated based on the average synonymous divergence between a randomly selected *X*. *gilli* sequence and an orthologus sequence from *X*. *tropicalis*, and assuming a divergence time of 65 million years for the separation of these lineages^63^, and a generation time of one year. Each locus had a mutation rate scalar based on synonymous divergence to *X*. *tropicalis* that accommodated variation among loci in the rate of evolution. To minimize the influence of natural selection on the polymorphism data, summary statistics and likelihood calculations were based only on variation at synonymous positions. Confidence intervals were estimated using the profile likelihood method (i.e., that the 95% confidence interval is defined by the two points that are 1.92 *log-likelihood (lnL)* units from the maximum).

### Population dynamics over time and space

We performed various analyses to assess whether the genetic diversity varied among these species, over time, or among populations east and west of False Bay. Pairwise *F*_*ST*_ (with significance computed by a permutation test) was quantified for the same data used in the STRUCTURE analysis using ARLEQUIN v3.5.2.2^64^. Nucleotide diversity (*π*) of each locus was calculated using the pegas package in R^65, 66^. We then calculated a mean value of *π* across loci for each of the eight populations, weighting the estimate by gene length for each locus. Confidence intervals were obtained by bootstrapping the weighted *π* values 5000 times.

Because allelic diversity is influenced by sample size, we using the program HP-RARE to calculate rarefied estimates of allelic diversity^67^, which involves downsampling data to the smallest number of samples in each population across all nuclear loci for which there were data. This analysis was performed with the same data as used in the STRUCTURE analysis. For *X*. *laevis* populations there was one exception; the *prmt6* locus had only four sampled alleles for the *X*. *laevis* west 1994 population, thus we did one run with all of the data (using four as the smallest number of sampled alleles) and another run excluding *prmt6* (in which case, eight was the smallest number of sampled alleles). For all *X*. *gilli* populations, the smallest number of sampled alleles was eight. We generated confidence intervals by bootstrapping of the allelic diversity measurements 5000 times.

To statistically evaluate differences in genetic diversity over time, location and species, we constructed linear mixed models using the R package lme4^68^. We built models for the estimated values of nucleotide diversity (*π*) and allelic diversity independently with diversity values measured for each locus, using time (1994 or 2014), location (east or west) and species (*X*. *gilli* or *X*. *laevis*) as fixed effects (all additive, no interaction terms) and considering locus as a random effect. For each parameter of both models, we also used lme4 to compute confidence intervals with the confint function.

## Results

### Molecular polymorphism and gene trees

**Figure 3:**
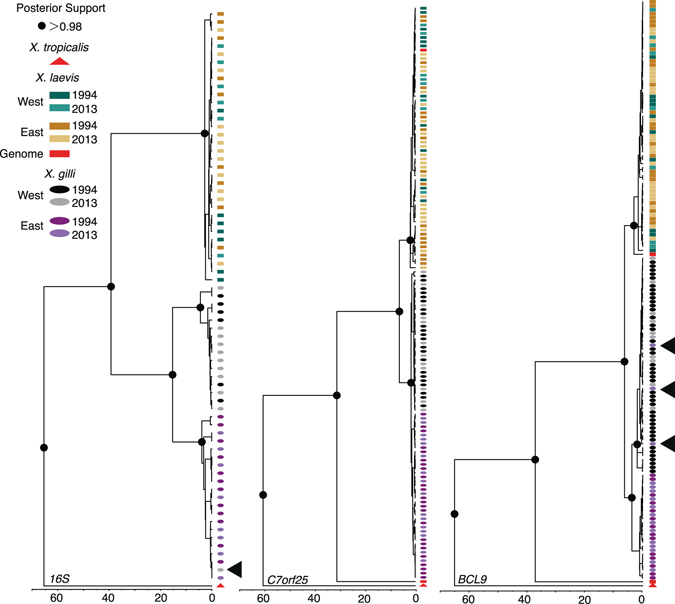
Representative gene trees that collectively provide no evidence of genetic exchange between *X*. *gilli* and *X*. *laevis*. The phylogeny on the left illustrates divergence between 16S rDNA mitochondrial sequences in the east and west populations of *X*. *gilli*, and with one shared sequence (indicated with an arrow) that occurred on both sides of False Bay. The nuclear phylogenies in the center and right provide examples of no shared alleles and shared alleles between the east and west *X*. *gilli* populations, respectively. Gene name acronyms are described in the Materials and Methods section. These and other phylogenies are depicted with sample labels in Fig. S1.

In the mitochondrial and 13 nuclear gene trees, alleles from *X*. *gilli* and *X*. *laevis* clustered in reciprocally monophyletic clades (Fig. 3, Fig. S1). No individuals were found to have introgressed loci, which would have been evidenced by an allele in one species having a closer relationship to the alleles of the other species (i.e. a paraphyletic relationship). Similar to previous studies^26, 27^, the mtDNA gene tree identified divergence between the east and west populations of *X*. *gilli* (Fig. 3). We identified one individual (Sample ID: XgUAE_08) from the west population of *X*. *gilli* that carried a mtDNA haplotype that was more closely related to haplotypes that were carried by individuals from the east population. This observation was also reported previously, from different samples^26, 27^. The *BEAST analysis recovered the expected species tree of these three species with posterior probabilities of one (Fig. S2). This analysis estimated the divergence time of *X*. *gilli* and *X*. *laevis* at about 14.05 my and divergenc of the east and west *X*. *gilli* populations at about 1 my (0.51–1.36 my 95% HDP; when a calibration point of 65 my from *X*. *tropicalis* is assumed^63^).

### Genetic clusters

**Figure 4:**
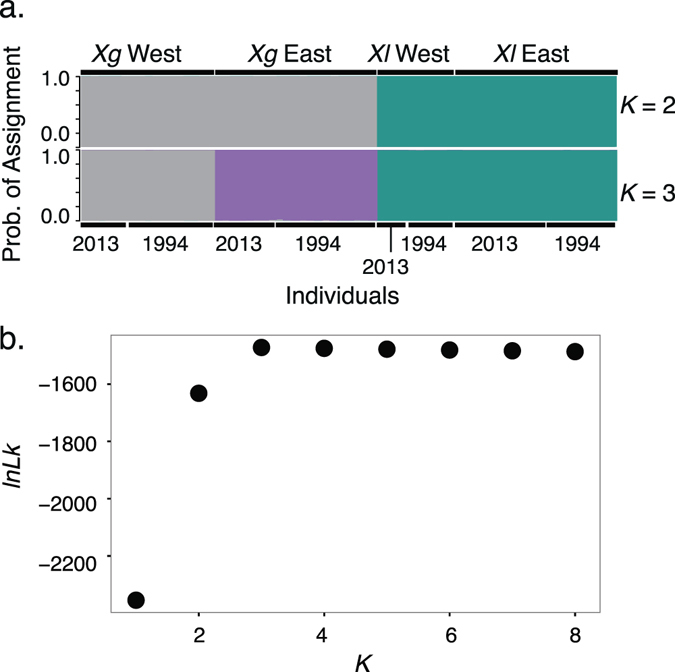
(**a**) Structure analyses for 10 loci, which had sequence data for all populations. (**b**) Likelihood for each value of *K*.

STRUCTURE analyses assigned each individual to groups that corresponded with species assignment (Fig. 4a). All *X*. *laevis* individuals were assigned to a single genetic cluster at *K* = 2–8, indicating a lack of allele frequency clustering, which is consistent with gene flow across the population range. The *X*. *gilli* samples were assigned to two clusters corresponding to sampling location (east and west) at *K* = 3–8, indicating differences in allele frequencies, which is consistent with restricted gene flow between them (Fig. 4a). Assignment of individuals to clusters stabilized at *K* = 3, with no new clusters being detected at higher values of *K* (Fig. 4a). The likelihood plot plateaus at *K* = 3 (Fig. 4b); the Evanno method^59^ supports *K* = 2 and the *ad hoc* method of ref. 57 supports *K* = 3.

### Evolutionary models

Using simulations and summary statistics, we evaluated the fit of the *X*. *gilli* data to evolutionary models with no migration after speciation, with ongoing migration after speciation, or with secondary contact after speciation. The *lnL* of the secondary contact model was −8.032, the ongoing migration model was −8.987, and the isolation model was <−13.815. We were not able to more precisely estimate the likelihood of the isolation model because no simulations under this model resulted in data whose four summary statistics were within ±*ε* of the observed values (see Methods).

Nested models can be compared by assuming that twice the difference between the *lnL* of each model follows a *χ* ^2^ distribution with degrees of freedom equal to the difference in the number of parameters in each model (denoted ${\chi }_{1}^{2}$ for comparison between models that differ in one parameter). However, because comparison between these successively more complex models involves a boundary condition on one parameter (*τ* = 1 for the ongoing migration model, *m* = 0 for the isolation model), this difference in model likelihoods follows a mixture of ${\chi }_{0}^{2}$ and ${\chi }_{1}^{2}$ distributions^69^. The secondary contact model is thus not supported over the ongoing migration model (*p* = 0.08), but the ongoing migration model is supported over the isolation model (*p* = 0.009). Overall then, these results support an inference of gene flow between *X. gilli* populations, but fail to discern substantial temporal heterogeneity in the level of gene flow.

The maximum likelihood parameter estimates and 95% confidence intervals for the ongoing migration model were *θ*: 0.002 (0.001–0.003) and *m*: 0.7 (0.1–2) individuals/generation. The maximum likelihood estimate for *T* was 8,500,000 generations; the 95% CI was unable to be estimated because it exceeded the boundaries we tested (1,000,000–20,000,000), suggesting low statistical power to estimate this parameter. Comparisons to similar parameters estimated for African clawed frogs by other studies using other methods^16, 36, 70^ suggest that these estimates are biologically plausible. Our intuition that the shared identical alleles between east and west *X*. *gilli* populations are due to ongoing migration is thus supported, with caveats that several model assumptions, discussed below, are violated to some degree.

### Population dynamics over time and space

In line with results from STRUCTURE analysis, a high *F*_*ST*_ was measured in all pairwise comparisons of the east and west populations of *X*. *gilli* (comparing within the same year 2013 east to 2013 west, and between years 1994 east to 2013 west and 2013 east to 1994 west; range: 0.55–0.60, *p* < 0.05). For *X*. *gilli*, between time points within each location (east or west), *F*_*ST*_ was not significantly different from zero (east 1994 to east 2013 and west 1994 to west 2013; *p* > 0.05, *F*_*ST*_ < 0.02). For *X*. *laevis*, pairwise comparisons of east and west populations, within the same year (1994 east to 1994 west or 2013 east to 2013 west) and between time points (1994 east to 2013 west and 2013 east to 1994 west), had intermediate *F*_*ST*_ values that departed significantly from zero (*p* < 0.05, *F*_*ST*_ = 0.07–0.16). But within locations comparing time points (1994 east to 2013 east and 1994 west to 2013 west), *F*_*ST*_ was not significantly different from zero (*p* > 0.05, *F*_*ST*_ = 0.04 for both comparisons).

**Figure 5:**
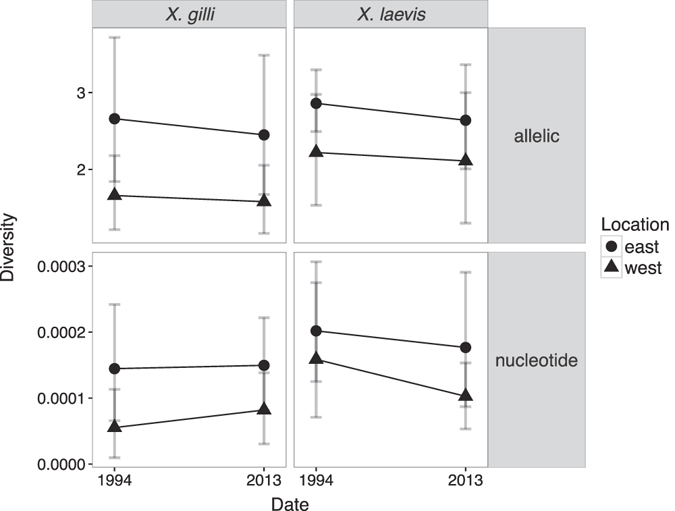
Genetic diversity statistics including rarefied estimates of allelic diversity (top panels) and nucleotide diversity (*π*) weighted by length of sequence; bottom panels). For allelic diversity, the analysis considered the same 10 loci as were analyzed by the STRUCTURE analysis (see Materials and Methods). Allelic diversity for *X*. *laevis* did not include *PRMT6* locus because this locus only had four alleles for the west 1994 population.

Both nucleotide diversity and allelic diversity did not change drastically over time, but within species, both statistics were higher in the east population than the west (Fig. 5). In the linear mixed model analysis of *π*, the effect of species was significant with *X*. *laevis* higher than *X*. *gilli* by 0.00073 substitutions per site (95% CI: 0.00027–0.00119). The effect of location was significant with *π* lower in the west than the east population by 0.00091 (95% CI: 0.00045–0.00137). The effect of time of sampling was not significant, with the 2013 samples being lower by 0.00016 but the 95% CI of th﻿is difference spanning zero (−0.00062–0.00030). Similar results were recovered for allelic diversity, with *X*. *laevis* having higher allelic diversity than *X*. *gilli* (0.59, 95% CI: 0.21–0.97), the west being less diverse than the east (−0.77, 95% CI: −1.14–−0.40), and no significant effect of sampling time (−0.15, 95% CI: −0.52–0.21).

## Discussion

### Gene Flow between *X*. *laevis* and *X*. *gilli*

Previous investigations of the genetic consequences of hybridization between *X*. *laevis* and *X*. *gilli* found no evidence of widespread genetic introgression^27, 28^, a result that seemed to be at odds with the incidence of morphologically and genetically identified hybrids in this and other studies^19, 26, 38, 39, 71^. In this study, we analyzed many of the samples from refs 27, 28 and also genetic samples that were collected more recently. Evidence of introgression between *X*. *laevis* and *X*. *gilli* was not detected in mitochondrial DNA or in any of 13 nuclear loci (Fig. 3; Fig. S1). Furthermore, each species formed separate genetic clusters with no evidence for similarities in allele frequencies (Fig. 4a). These findings were consistent in both sampling efforts examined here, which included targeting both populations of *X*. *gilli* and sampling time points separated by about two decades. Previous investigations into the extent of genetic introgression^28^, used two nuclear loci that were not used in this study. Combining that study with ours brings the total number of genomic regions studied to 15, and includes variation from 6 of the 18 chromosome pairs based on gene location in the *X*. *laevis* version 9 genome, on Xenbase. This expanded sampling is thus consistent with the interpretation by ref. 28 that genomic introgression is not extensive.

The lack of introgression is despite the continued identification (based on morphology) of a low frequency of putative *F*_1_ hybrids in both localities. Though there could be an adaptive benefit for hybridization because  *X*. *gilli* embryos can tolerate ponds with higher pH levels than *X*. *laevis*, which perhaps could allow  for invasion of *X*. *gilli* habitat, we found no evidence that hybridization has led to gene flow of the genetic basis of this or other ecological adaptations that evolved after these two species diverged from their most recent common ancestor. Although not the focus of this study, the relatively low abundance of *F1* hybrids argues against the possibility that a new species of hybrid origin is evolving in this zone of sympatry between *X*. *laevis* and *X*. *gilli*. Reproductive isolation in amphibians has been shown to happen in a few million years for some lineages^72, 73^. In *Xenopus* species, female individuals respond to species-specific calls evoked by males (phonotaxis)^74^ and this presumably acts to some degree as a prezygotic barrier to hybridization. However, an observation is that at high densities, *Xenopus* individuals amplex indiscriminately (G. J. Measey, personal observation), potentially overriding some prezygotic barriers. In some ponds, *X*. *gilli* individuals can be outnumbered by *X*. *laevis* 3:1^41^, and indiscriminate amplexus could mean *X*. *gilli* males (which are also smaller) are outcompeted for access to females. This may be why hybrids are occasionally seen, but the extended period of divergence between these species (~14 my) appears to have resulted in strong post-zygotic barriers preventing introgression.

Hybridization followed by back-crossing is expected to generate a mosaic of introgressed and non-introgressed genomic regions. Variation among genomic regions in the extent of introgression can be further augmented by natural selection favoring or disadvantaging genetic variants from one species in the genomic background of the other^5^. In California tiger salamanders (*Ambystoma californiense*), for example, some loci are fixed for foreign alleles from the introduced barred tiger salamander (*A*. *mavortium*), whereas other loci exhibit no sign of introgression^6^. That the barred tiger salamander was introduced only 60 years ago suggests that mosaicism of genomic introgression arose rapidly (in ~20 generations; ref. 6). In this study it is therefore possible that we failed to identify some introgressed regions of the genome due to the relatively sparse sampling of genomic regions. Future studies that survey variation across the entire genome, such as RAD-Seq^75^, could more precisely quantify the extent of gene flow between these species, if it occurs.

### Population structure in *X*. *gilli* and change over time

Analysis of mtDNA^26, 27, 45^ and skin peptides secreted by these populations^76^ support the existence of at least two distinct populations in *X*. *gilli* in the western and eastern portion of its range. Our mitochondrial analysis, STRUCTURE analysis, and some of the gene trees reported here (such as *MOGS*-*1* and *RASSF10*) also exhibit substantial geographic differences in *X*. *gilli* allele frequencies between these populations (Figs 3 and 4a, Fig. S1). In contrast, genetic diversity in *X*. *laevis* has minimal geographic structure, with most alleles occurring on both sides of False Bay, and STRUCTURE analyses assigning all *X*. *laevis* individuals to a single genetic cluster (Fig. S1, Fig. 4a). This is similar to findings reported by ref. 27.

When and why did population structure arise in *X*. *gilli*? Using mtDNA sequence data and a relaxed molecular clock^45^, estimated that the divergence between *X*. *gilli* populations occurred 8.5 million years (my) (95% CI: 4.8–13.4), which is the same as the estimate obtained here using our coalescent modeling approach. This estimate is older than the 1 my divergence time estimated by the *BEAST analysis (Fig. S2), but this is not unexpected because *BEAST does not incorporate gene flow after divergence in its model. Using similar data and a coalescent modeling approach ref. 26 recovered a somewhat more recent divergence time of 4.63 my, but with confidence intervals that overlapped with the previous estimate (95% CI: 3.17–6.38). Evans *et al.* ^27^ proposed that the two populations split following inundation of the Cape Flats. Fogell *et al.* ^26^ pointed out that marine inundation probably occurred multiple times in the last few million years and that cycles of aridification also likely influenced the costal fynbos habitat, on which *X*. *gilli* relies. Our finding of gene flow after divergence supports the idea that these populations have been periodically reconnected, allowing exchange of migrants. Therefore, whatever the cause of divergence was, it was demonstrably not a permanent barrier.

Of note is that the evolutionary models we tested are almost certainly violated by the system we explored in many ways, including variation over time and among populations in population size, mutation rate, and migration rate. Although we do not anticipate that these violations are influential enough as to negate the rejection of the isolation model, a larger dataset might provide statistical power with which to better evaluate more complex scenarios, such as the secondary contact model.

The *F*_*ST*_ and linear mixed model analyses suggest that allele frequencies have not changed substantially in the last 20 years, though there is a trend of decreasing diversity (Fig. 5 and from values obtained in linear mixed models indicated a non- significant decline in diversity from 1994 to 2013). If generation time is about one year or less (which is based on laboratory studies and could be an underestimate, ref. 35), this represents 20 generations. Changes in allelic diversity may signal population declines earlier than nucleotide diversity, because loss of rare alleles (which happens during population declines) would have a greater impact on count based metrics, such as allelic diversity, than they would on frequency based metrics such as *π* ^77^. Thus, though not significant, a declining trend seen for allelic diversity (Fig. 5) may be an early indication of population declines. Linear mixed models allowing for independent changes in diversity for each locus over time revealed declining genetic diversity (except for two loci in the *π* models; results not shown).

### Management

Hybridization and introgression has the potential to threaten species survival^10^. In an attempt to reduce gene flow between species, three conservation actions were implemented in the mid-1980s. A wall was built around one impoundment in CoGH^25^, populations of pure *X*. *gilli* were translocated to areas without *X*. *laevis* ^40^, and *X*. *laevis* were manually removed from CoGH^25, 41, 78^. Removal of *X*. *laevis* ceased in 2000, but resumed in 2011^25, 41, 78^. The same management efforts have not been conducted for the population of *X*. *gilli* east of False Bay, most of which resides in non-protected areas^26, 41^.

Interestingly, the CoGH has greater juvenile recruitment of *X*. *gilli* ^41^ and fewer hybrids (2.5% vs 8–27% of individuals in ponds in the west and east respectively, ref. 26). Our results suggest that these hybrids are not producing successful offspring via backcrossing with either parental species frequently enough to produce large scale genomic impacts. These results suggest that the genomes of *X*. *gilli* and *X*. *laevis* are largely genetically distinct. Thus, the major benefits to *X*. *gilli* of removal of *X*. *laevis* from habitat shared with *X*. *gilli* probably stem from minimizing competition for ecological resources between these species^25, 41, 42^.

For *X*. *gilli* and *X*. *laevis*, east populations harbor more genetic diversity than the west populations (Fig. 5). Allelic diversity and heterozygosity reflect a population’s ability to respond to selection^79^, and thus from a genetic perspective conservation of east populations of *X*. *gilli* is paramount.

This study suggests that patterns of gene flow within *X*. *gilli* included genetic exchange between populations in the east and west. The ancestral distribution of *X*. *gilli* was likely patchy to begin with and has contracted considerably in the last several decades, including in locations now occupied only by *X*. *laevis* or *X*. *laevis* and hybrids^25, 26^. Ancestral patterns of gene flow are presumably imperiled by further habitat fragmented by human activity, including habitat altering effects of invasive species such as *Acacia saligna* (Port Jackson Willow), *Acacia mearnsii* (Black Wattle), and *Hakea sericea* (Silky Hakea)^80^. Continued efforts to conserve and restore coastal fynbos habitat both inside and outside of protected areas^43^, such as removal of invasive vegetation, restoration of native vegetation, and removal of *X*. *laevis*, stands to benefit *X*. *gilli*. This is particularly important in the east population of *X*. *gilli* near Kleinmond where genetic diversity is highest and the population resides on private land.

## Electronic supplementary material

Supplementary Table 1

Supplemental Figures 1 and 2
