# Supplementary material for: Limited genomic consequences of hybridization between two African clawed frogs, Xenopus gilli and X. laevis (Anura: Pipidae)
Source: Sci Rep. 2017 Apr 24;7:1091. doi: 10.1038/s41598-017-01104-9 (PMC5430669; doi:10.1038/s41598-017-01104-9)
Supplement: Supplementary file 1 — Supplementary Table 1 [file 41598_2017_1104_MOESM1_ESM.pdf]

Supplementary Table 1. Sample IDs and loci sequenced for each individual in this study. Locus acronyms are defined in the main text. A 1 or a 0 indicats that the gene was or was not sequenced sequenced respectively.

Sample_ID	16s	btbd	c7or	fem1	mas	mog	nfil1	pcdh	pigo	prm	rass	sugp	zbec	bcl9
XgUAE_11_gilli_WestCape_2013	1	1	1	1	1	1	1	0	0	1	1	1	1	1
XgUAE_02_gilli_WestCape_2013	1	0	0	0	0	0	0	0	0	0	0	0	0	0
XgUAE_66_gilli_WestCape_2013	1	1	1	1	1	1	1	1	0	1	1	1	1	1
XgUAE_03_gilli_WestCape_2013	1	0	0	0	0	0	0	0	0	0	0	0	0	0
XgUAE_06_gilli_WestCape_2013	1	1	1	1	1	0	1	1	0	0	1	1	1	1
XgUAE_01_gilli_WestCape_2013	1	0	0	0	0	0	0	0	0	0	0	0	0	0
XgUAE_05_gilli_WestCape_2013	1	0	1	1	0	1	1	1	0	1	1	1	1	1
XgUAE_07_gilli_WestCape_2013	1	1	1	1	1	1	1	0	0	1	1	1	1	0
XgUAE_04_gilli_WestCape_2013	1	0	0	0	0	0	0	0	0	0	0	0	0	0
XgUAE_08_gilli_WestCape_2013	1	1	1	1	1	0	1	1	0	1	1	1	1	1
Xb_1_2_gilli_WestCape_1994	0	0	0	0	0	0	0	0	0	0	0	0	0	1
Xb_12_13_gilli_WestCape_1994	0	0	0	0	0	0	0	0	0	0	0	0	0	1
Rg_0_1_gilli_WestCape_1994	1	0	0	0	0	0	0	0	0	0	0	0	0	0
Rg_2_gilli_WestCape_1994	0	1	1	0	0	1	1	0	0	1	0	1	1	0
Rg_1_2_0_gilli_WestCape_1994	1	0	0	0	0	0	0	0	0	0	0	0	0	0
Rg_3_1_gilli_WestCape_1994	0	0	0	0	0	0	0	0	0	0	0	0	0	1
Xb_12_1_gilli_WestCape_1994	0	1	1	1	1	1	1	0	0	1	1	1	1	1
Rg_2_1_gilli_WestCape_1994	0	1	1	1	1	1	1	0	0	1	1	1	1	1
Rg_1_1_gilli_WestCape_1994	1	0	0	0	0	0	0	0	0	0	0	0	0	0
Jd_1_2_gilli_WestCape_1994	0	1	1	1	1	1	1	1	0	0	1	1	1	1
Xg_123_3_gilli_WestCape_1994	0	1	1	1	1	1	1	1	0	1	1	1	1	1
Xg_23_1_gilli_WestCape_1994	0	0	0	0	0	0	0	0	0	0	0	0	0	1
Rg_1_3_gilli_WestCape_1994	0	1	1	1	1	1	1	1	0	1	1	1	1	1
Jd_0_2_gilli_WestCape_1994	0	1	1	0	0	1	1	1	0	1	1	1	1	1
Rg_1_gilli_WestCape_1994	0	1	0	0	0	1	1	0	0	1	0	1	1	0
Xb_2_3_gilli_WestCape_1994	0	0	0	0	0	0	0	0	0	0	0	0	0	1
Sd_3_2_gilli_WestCape_1994	1	0	0	0	0	0	0	0	0	0	0	0	0	0
Sd_2_0_gilli_WestCape_1994	0	1	1	1	1	1	1	1	0	1	1	1	1	1
Sd_1_3_gilli_WestCape_1994	0	1	1	1	1	1	1	1	0	1	1	1	1	1
Rg_0_3_gilli_WestCape_1994	0	0	0	0	0	0	0	0	0	0	0	0	0	1
Jd_3_0_gilli_WestCape_1994	0	0	0	0	0	0	0	0	0	0	0	0	0	1
Jd_1_0_gilli_WestCape_1994	0	0	0	0	0	0	0	0	0	0	0	0	0	1
Rg_2_3_gilli_WestCape_1994	0	0	0	0	0	0	0	0	0	0	0	0	0	1
Sd_0_1_gilli_WestCape_1994	0	0	0	0	0	0	0	0	0	0	0	0	0	1
Xg_3_2_gilli_WestCape_1994	0	1	1	1	1	1	1	1	0	1	1	1	1	1
Rg_0_2_gilli_WestCape_1994	1	0	0	0	0	0	0	0	0	0	0	0	0	0
Xb_3_0_gilli_WestCape_1994	0	1	1	1	1	1	1	1	0	1	1	1	1	1
Sd_3_1_gilli_WestCape_1994	1	0	0	0	0	0	0	0	0	0	0	0	0	1
XgUAE_132_gilli_EastCape_2013	1	1	1	1	1	1	1	1	0	1	1	1	1	1
XgUAE_133_gilli_EastCape_2013	1	1	1	1	1	1	1	1	0	1	1	1	1	1
XgUAE_128_gilli_EastCape_2013	1	1	1	1	1	1	1	1	0	1	1	1	1	1
XgUAE_134_gilli_EastCape_2013	1	1	1	1	1	1	1	1	0	1	1	1	1	1

XgUAE_130_gilli_EastCape_2013	1	1	1	0	1	1	1	1	0	1	1	1	1	1
XgUAE_129_gilli_EastCape_2013	1	1	1	1	1	1	1	1	0	1	1	1	0	1
XgUAE_127_gilli_EastCape_2013	1	1	1	1	1	1	1	1	0	1	1	1	1	1
XgUAE_131_gilli_EastCape_2013	1	1	1	1	1	1	1	1	0	1	1	1	1	1
Xr_1_2_gilli_EastCape_1994	0	1	1	1	1	0	1	0	0	1	1	1	1	1
Xm_3_0_gilli_EastCape_1994	0	1	1	1	1	0	1	0	0	1	1	1	0	1
Xm_0_2_gilli_EastCape_1994	0	0	1	1	1	0	1	0	0	1	1	1	1	0
Xr_31_2_gilli_EastCape_1994	0	1	1	1	1	0	1	0	0	0	1	1	1	0
Xr_1_1_gilli_EastCape_1994	1	1	0	1	1	0	1	0	0	1	1	1	1	1
Xr_12_3_gilli_EastCape_1994	1	0	0	0	0	0	0	0	0	0	0	0	0	0
Xr_0_3_gilli_EastCape_1994	1	1	1	1	1	0	1	0	0	1	1	1	1	1
Xr_2_2_gilli_EastCape_1994	0	1	1	1	1	0	1	0	0	0	1	1	1	0
Xm_2_1_gilli_EastCape_1994	1	0	0	0	0	0	0	0	0	0	0	0	0	0
Xm_2_0_gilli_EastCape_1994	0	0	0	0	0	0	0	0	0	0	0	0	0	1
Xr_2_1_gilli_EastCape_1994	1	1	1	1	1	0	1	0	0	1	1	1	1	1
Xr_3_1_gilli_EastCape_1994	0	1	1	1	1	0	1	0	0	1	1	1	1	0
Km_01_3_gilli_EastCape_1994	1	0	0	0	0	0	0	0	0	0	0	0	0	0
Km_2_gilli_EastCape_1994	1	1	1	1	1	0	0	0	0	1	1	1	1	0
Xr_3_2_gilli_EastCape_1994	1	0	0	0	0	0	0	0	0	0	0	0	0	0
Km_1_gilli_EastCape_1994	0	1	1	1	1	0	1	0	0	1	1	1	0	0
Xs_92_gilli_EastCape_1994	1	0	0	0	0	0	0	0	0	0	0	0	0	0
Xr_12_1_gilli_EastCape_1994	1	0	0	0	0	0	0	0	0	0	0	0	0	0
Xr_3_12_gilli_EastCape_1994	1	0	0	0	0	0	0	0	0	0	0	0	0	0
Xr_2_12_gilli_EastCape_1994	1	0	0	0	0	0	0	0	0	0	0	0	0	0
Xm_0_1_gilli_EastCape_1994	0	1	1	1	1	0	1	0	0	1	1	1	1	0
Xr_16_gilli_EastCape_1994	1	1	1	1	1	0	1	0	0	1	1	1	1	1
XgUAE_62_laevis_WestCape_2013	1	0	0	0	0	0	0	0	0	0	0	0	0	0
XgUAE_65_laevis_WestCape_2013	1	1	1	1	1	1	1	1	0	1	1	1	1	1
XgUAE_68_laevis_WestCape_2013	1	1	1	1	1	1	1	1	0	1	1	1	1	1
XgUAE_63_laevis_WestCape_2013	1	1	1	1	1	1	1	1	0	1	1	1	1	1
XgUAE_60_laevis_WestCape_2013	1	0	0	0	0	0	0	0	0	0	0	0	0	0
XgUAE_69_laevis_WestCape_2013	1	1	1	1	1	1	1	1	0	1	1	1	1	1
Lg_12_0_laevis_WestCape_1994	1	0	0	0	0	0	0	0	0	0	0	0	0	0
Lg_2_1_laevis_WestCape_1994	0	0	1	0	1	1	1	0	0	1	1	1	1	0
Lg_1_1_laevis_WestCape_1994	0	1	1	1	1	1	1	1	0	1	1	1	1	1
Lg_2_3_laevis_WestCape_1994	1	0	0	0	0	0	0	0	0	0	0	0	0	0
Rgl_3_laevis_WestCape_1994	0	1	1	1	1	0	1	0	0	0	1	1	1	1
Xl_28_laevis_WestCape_1994	1	0	0	0	0	0	0	0	0	0	0	0	0	0
Rgl_1_laevis_WestCape_1994	1	1	1	1	1	0	1	0	0	0	1	1	1	1
Rgl_2_laevis_WestCape_1994	0	1	1	1	1	1	1	1	0	0	1	1	1	1
Lg_2_2_laevis_WestCape_1994	0	1	1	1	1	0	0	0	0	0	1	1	1	1
Lg_12_38_laevis_WestCape_1994	1	0	0	0	0	0	0	0	0	0	0	0	0	0
Xl_2_0_laevis_WestCape_1994	1	0	0	0	0	0	0	0	0	0	0	0	0	0
XgUAE_104_laevis_EastCape_2013	1	1	1	1	1	1	1	0	0	1	1	1	0	1
XgUAE_124_laevis_EastCape_2013	0	0	0	0	0	0	0	1	0	0	0	0	0	0

XgUAE_102_laevis_EastCape_2013	1	1	1	1	1	1	1	0	0	1	1	1	1	1
XgUAE_123_laevis_EastCape_2013	1	0	1	0	1	1	1	0	0	1	1	0	1	1
XgUAE_105_laevis_EastCape_2013	1	0	1	1	1	1	1	1	0	1	1	1	1	1
XgUAE_101_laevis_EastCape_2013	1	1	1	1	1	1	1	0	0	1	1	1	1	1
XgUAE_109_laevis_EastCape_2013	1	1	1	1	1	1	1	1	0	1	1	1	1	1
XgUAE_108_laevis_EastCape_2013	1	1	1	0	1	1	1	0	0	1	1	1	1	1
XgUAE_103_laevis_EastCape_2013	1	1	1	1	1	1	1	0	0	1	1	1	1	1
XgUAE_106_laevis_EastCape_2013	1	1	1	1	1	1	1	0	0	1	1	1	1	1
XgUAE_107_laevis_EastCape_2013	1	1	1	1	1	1	1	1	0	1	1	1	1	1
XgUAE_124_laevis_EastCape_2013	1	1	1	1	1	1	1	0	0	1	1	1	1	1
XgUAE_100_laevis_EastCape_2013	1	1	1	1	1	1	1	1	0	1	1	1	1	1
Ea_12_1_laevis_EastCape_1994	0	0	0	0	0	0	0	0	0	1	0	0	0	0
Kml_8_laevis_EastCape_1994	1	1	1	1	1	1	1	0	0	1	1	1	1	1
Xsl_18_laevis_EastCape_1994	0	1	0	0	0	0	1	0	0	0	0	1	1	0
Xsl_15_laevis_EastCape_1994	0	1	1	0	1	1	1	0	0	1	1	1	1	1
Xsl_5_laevis_EastCape_1994	1	1	1	1	1	1	1	0	0	1	1	1	1	1
Kml_6_laevis_EastCape_1994	1	1	1	1	1	1	1	0	0	1	1	1	1	1
Ea_7_laevis_EastCape_1994	1	1	1	0	1	1	1	0	0	1	1	1	1	0
Ea_6_laevis_EastCape_1994	0	1	1	1	1	1	1	0	0	1	1	1	0	1
Xsl_3_laevis_EastCape_1994	1	0	0	0	0	1	0	0	0	1	0	0	0	1
Ea_4_laevis_EastCape_1994	1	0	0	0	0	1	0	0	0	1	0	0	0	0
Xsl_4_laevis_EastCape_1994	1	1	1	0	1	0	1	0	0	0	1	1	1	1
Kml_5_laevis_EastCape_1994	1	0	0	0	0	1	0	0	0	1	0	0	0	1
Xsl_7_laevis_EastCape_1994	0	0	0	0	0	0	0	0	0	0	0	0	0	1
Ea_5_laevis_EastCape_1994	0	1	1	1	1	0	1	0	0	0	1	1	1	1
Kml_7_laevis_EastCape_1994	1	1	1	1	1	1	1	0	0	1	1	1	1	1
