# Supplementary material for: Limited genomic consequences of hybridization between two African clawed frogs, Xenopus gilli and X. laevis (Anura: Pipidae)
Source: Sci Rep. 2017 Apr 24;7:1091. doi: 10.1038/s41598-017-01104-9 (PMC5430669; doi:10.1038/s41598-017-01104-9)
Supplement: Supplementary file 2 — Supplemental Figures 1 and 2 [file 41598_2017_1104_MOESM2_ESM.pdf]

Supplemental Figures:

Limited genomic consequences of hybridization between two African clawed frogs, *Xenopus gilli* and *X. laevis* (Anura: Pipidae)

Benjamin L. S. Furman[1], **Caroline M. S. Cauret**[1], **Graham A. Colby**[1], **G. John Measey**[2], **and Ben J. Evans**[1,2,*]

[1]Biology Department, Life Sciences Building room 328, McMaster University, 1280 Main Street West, Hamilton, ON L8S 4K1 Canada
[2]Centre for Invasion Biology, Department of Botany and Zoology, Stellenbosch University, Private Bag X1, Matieland 7602, Stellenbosch, South Africa
[*]evansb@mcmaster.ca

ABSTRACT

The Cape platanna, *Xenopus gilli*, an endangered frog, hybridizes with the African clawed frog, *X. laevis*, in South Africa. Estimates of the extent of gene flow between these species range from pervasive to rare. Efforts have been made in the last 30 years to minimize hybridization between these two species in the west population of *X. gilli*, but not the east populations. To further explore the impact of hybridization and the efforts to minimize it, we examined molecular variation in one mitochondrial and 13 nuclear genes in genetic samples collected recently (2013/4) and also over two decades ago (1994). Despite the presence of F_1 hybrids in the east, none of the genomic regions we surveyed had evidence of gene flow between these species, indicating a lack of extensive introgression. Additionally we found no significant effect of sampling time on genetic diversity of populations of each species. Thus, we speculate that F_1 hybrids have low fitness and are not backcrossing with the parental species to an appreciable degree. Within *X. gilli*, evidence for gene flow was recovered between western and eastern populations, a finding that has implications for conservation management of this species and its threatened habitat.

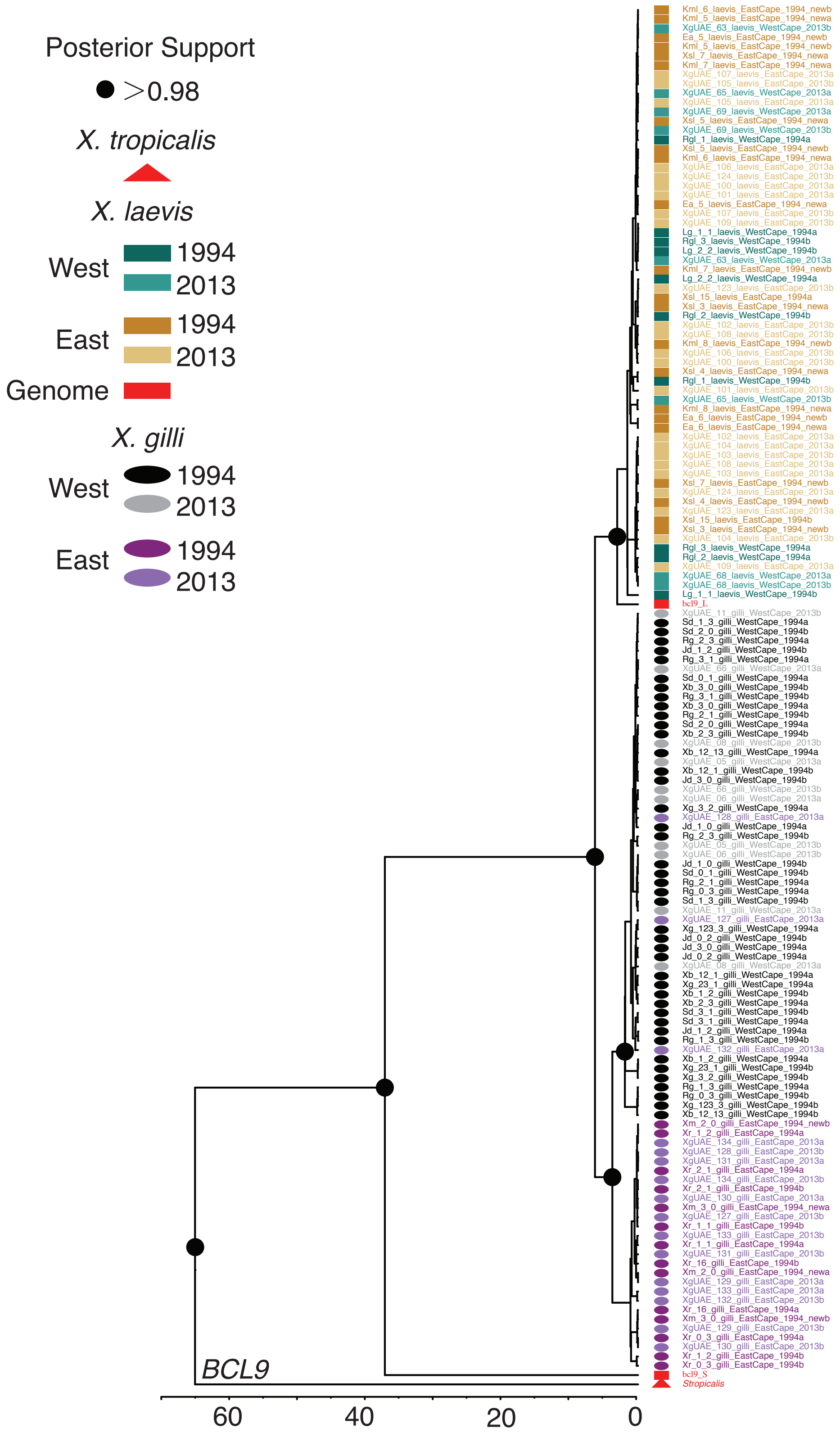

Figure S1. Phylogenetic trees for all loci included in this study. Sample names included at tip labels.

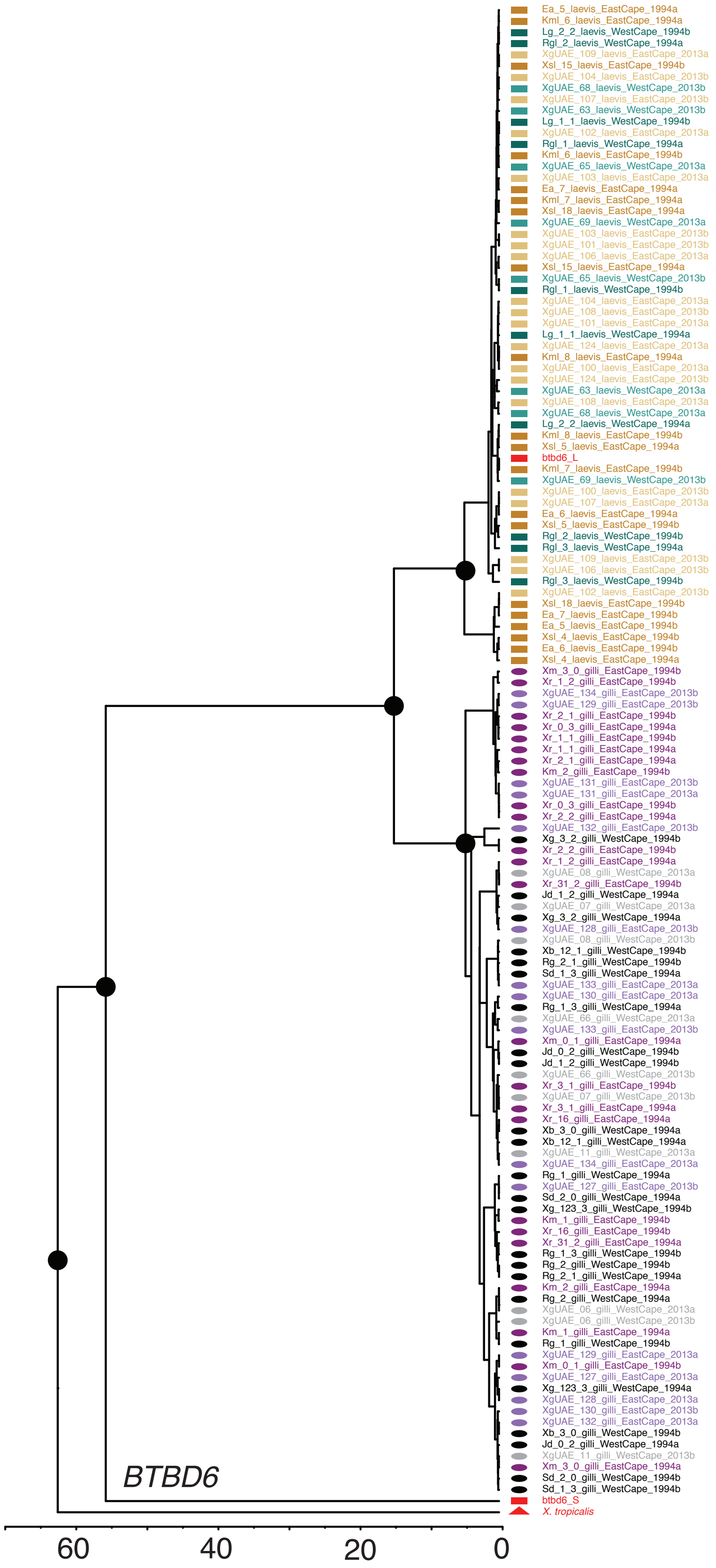

Figure S1. continued.

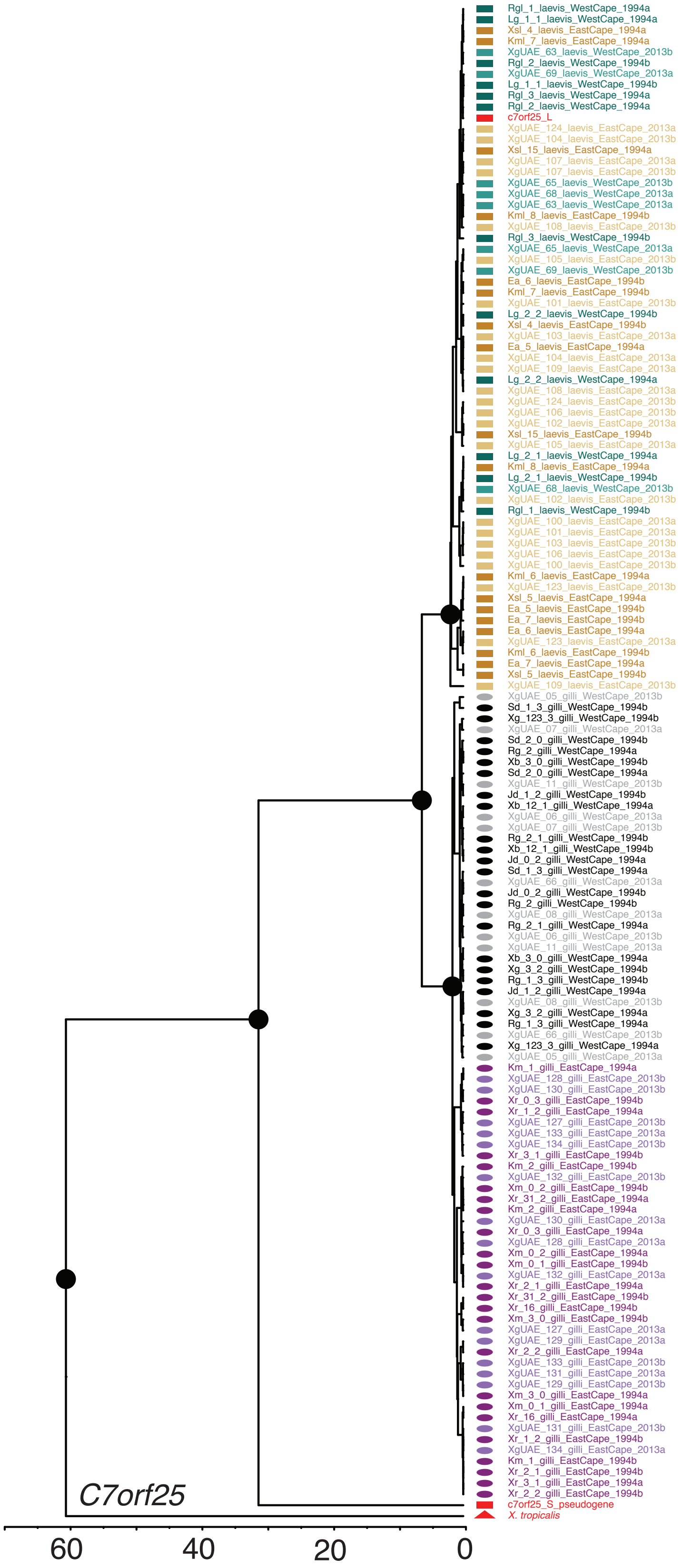

Figure S1. continued.

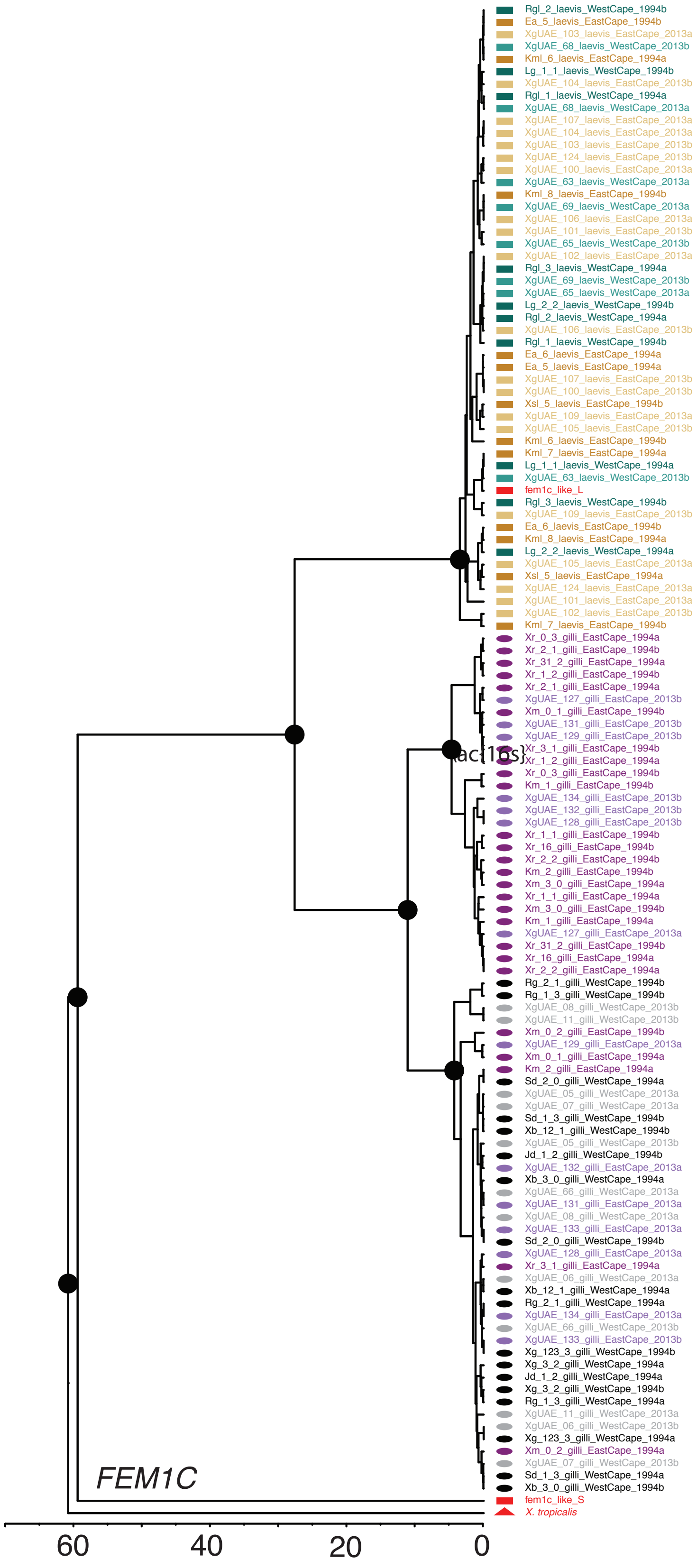

Figure S1. continued.

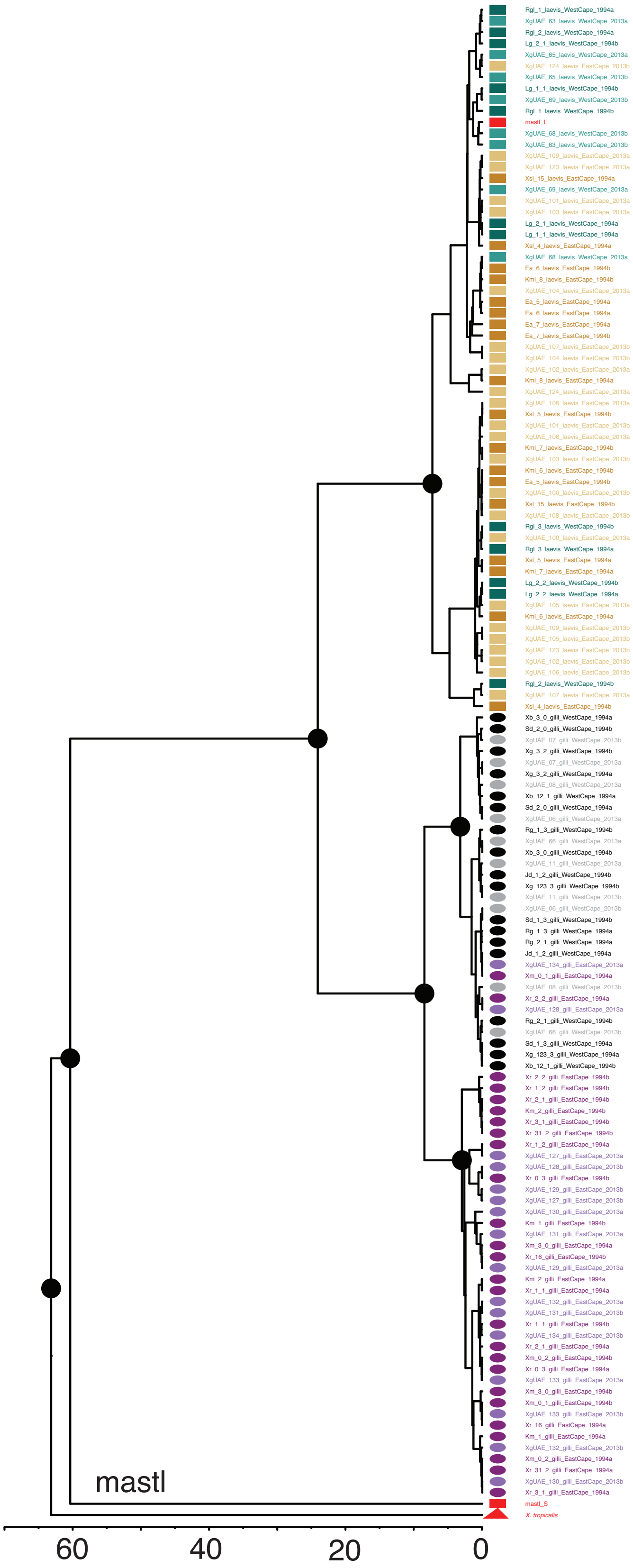

Figure S1. continued.

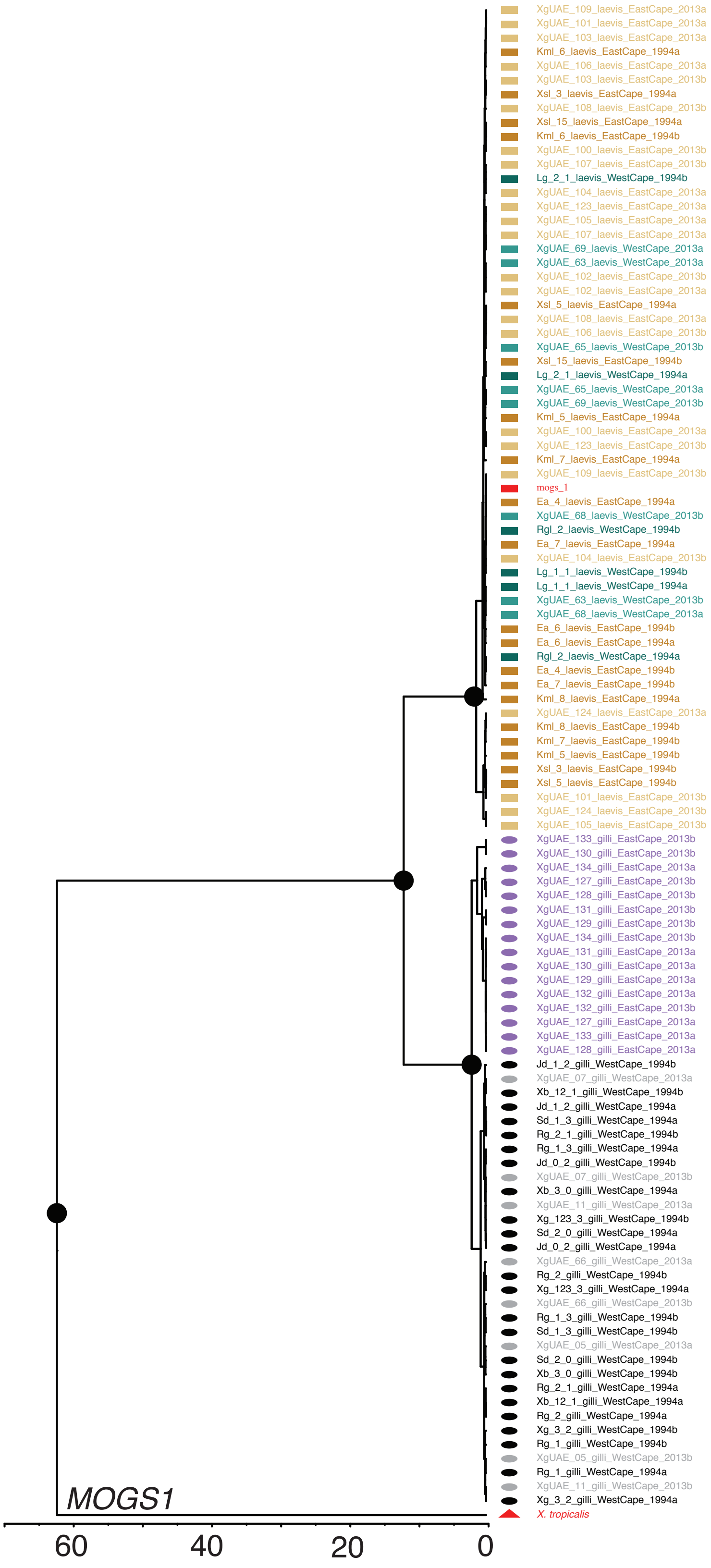

Figure S1. continued.

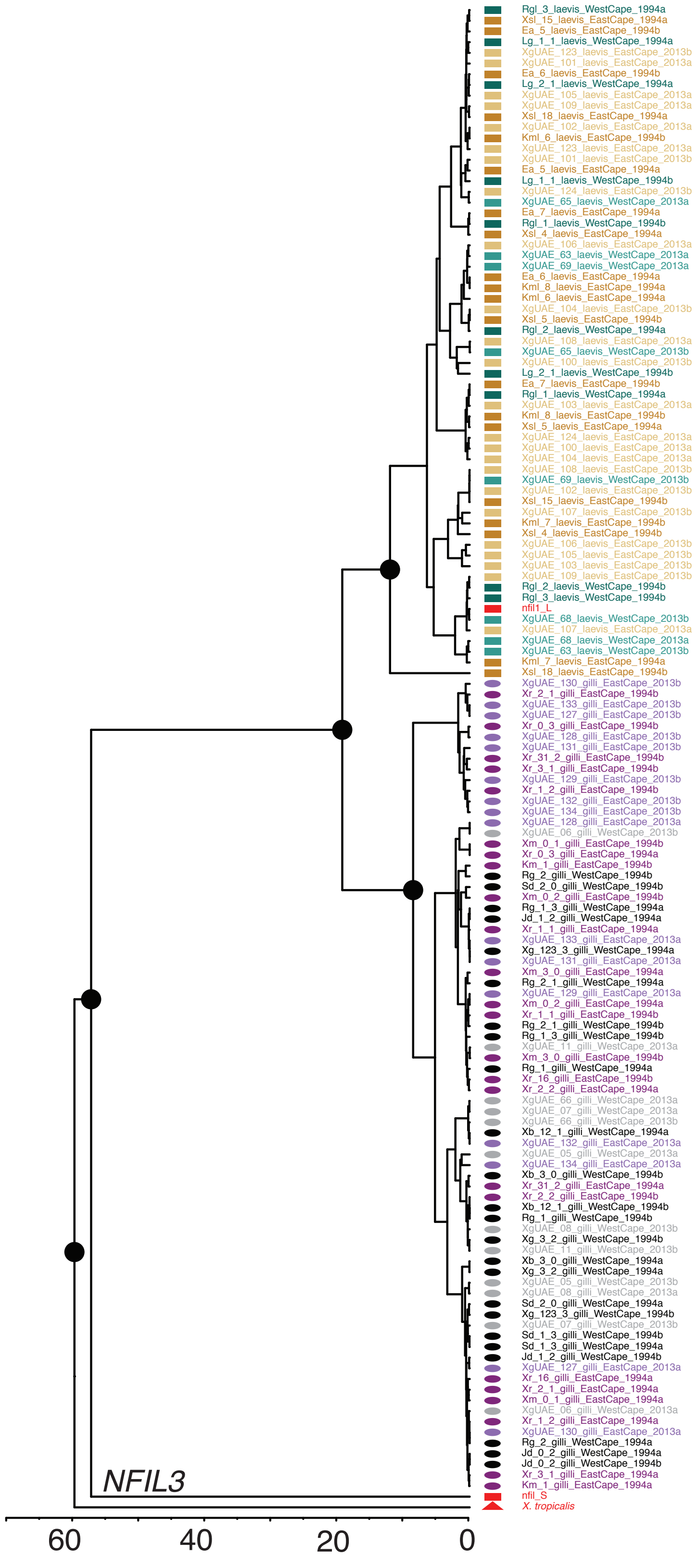

Figure S1. continued.

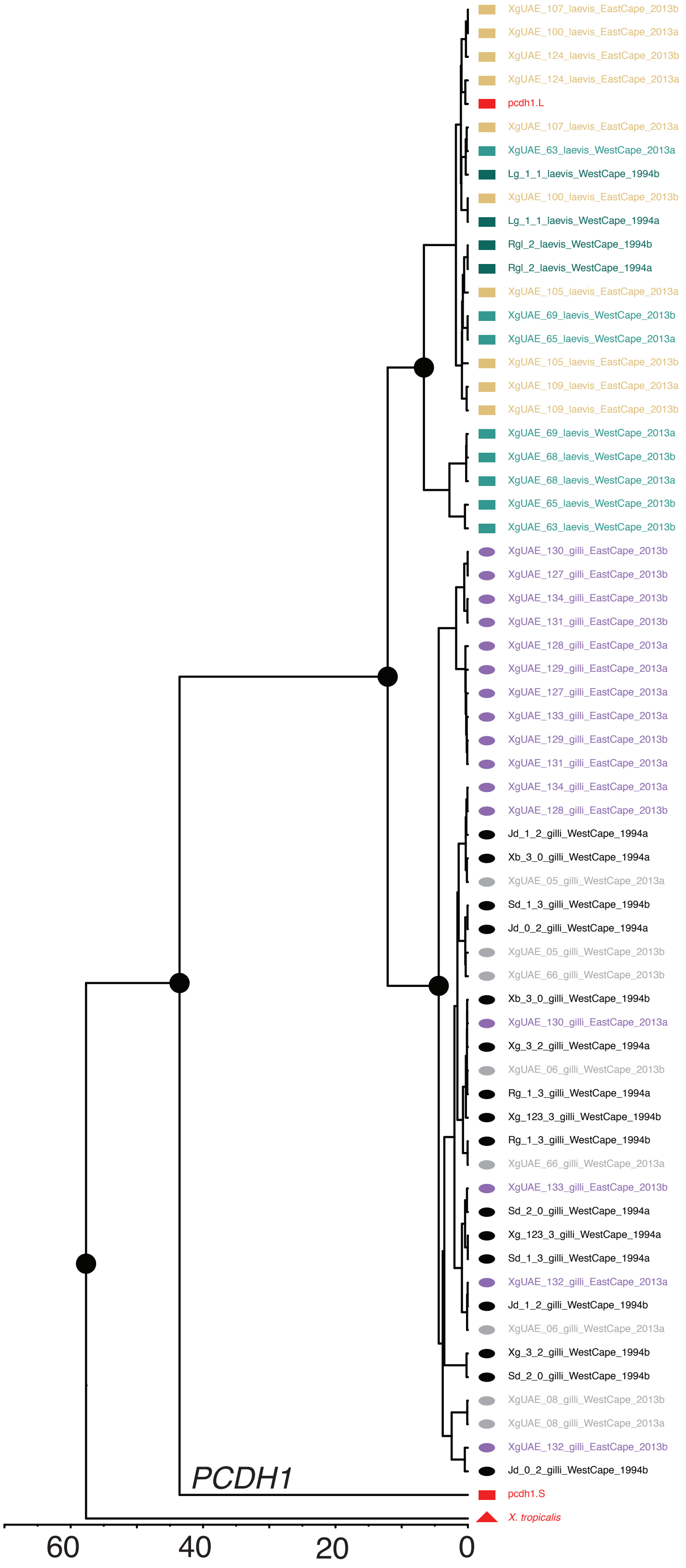

Figure S1. continued.

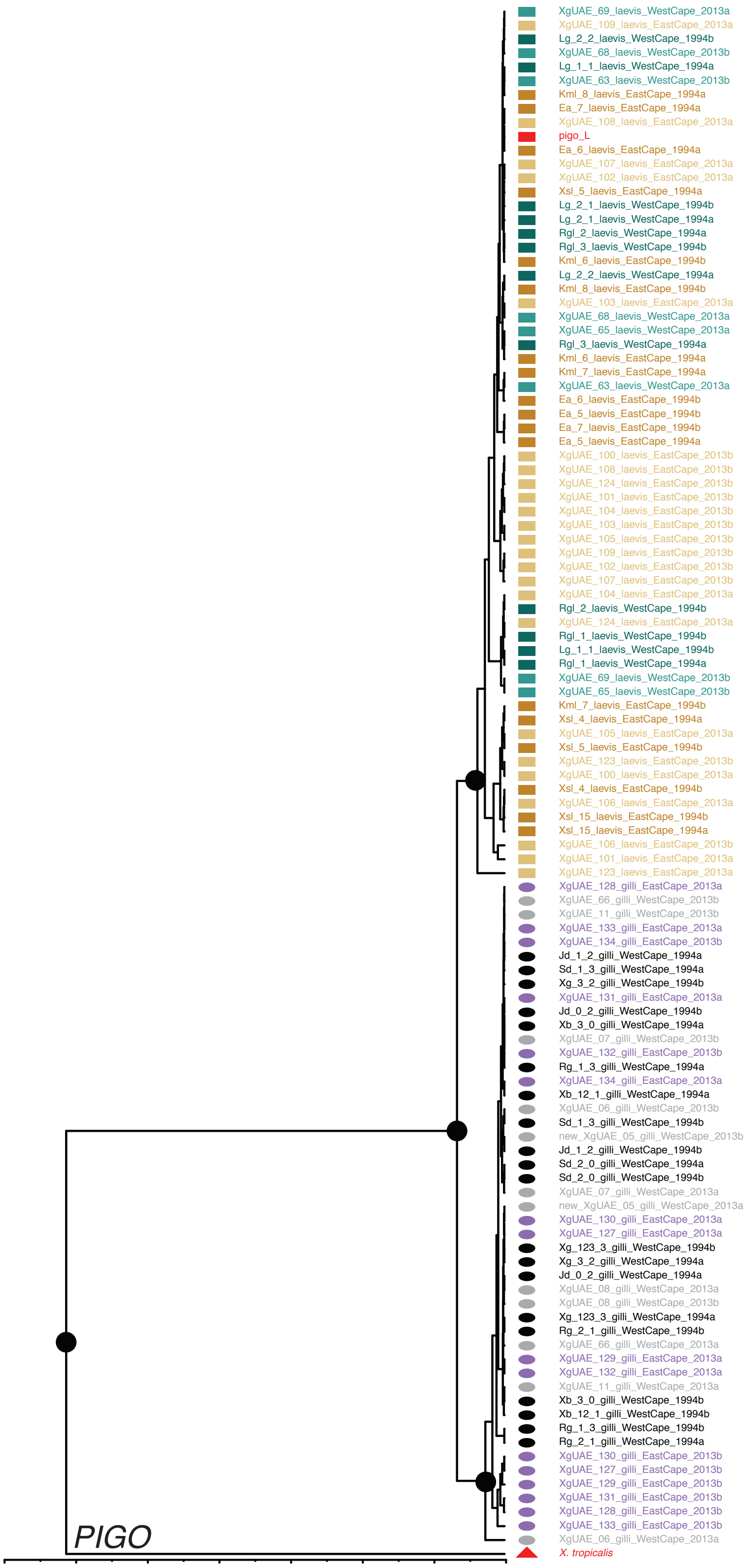

Figure S1. continued.

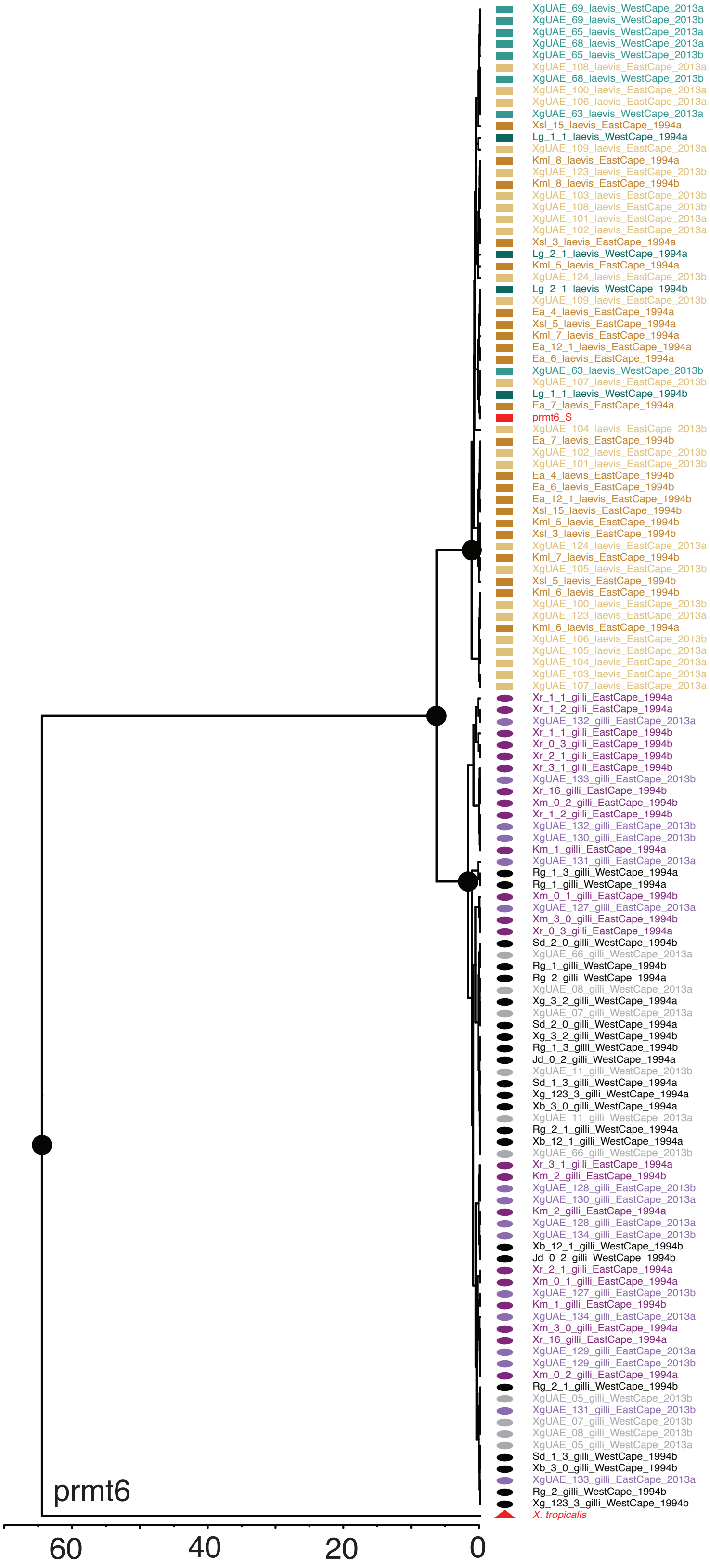

Figure S1. continued.

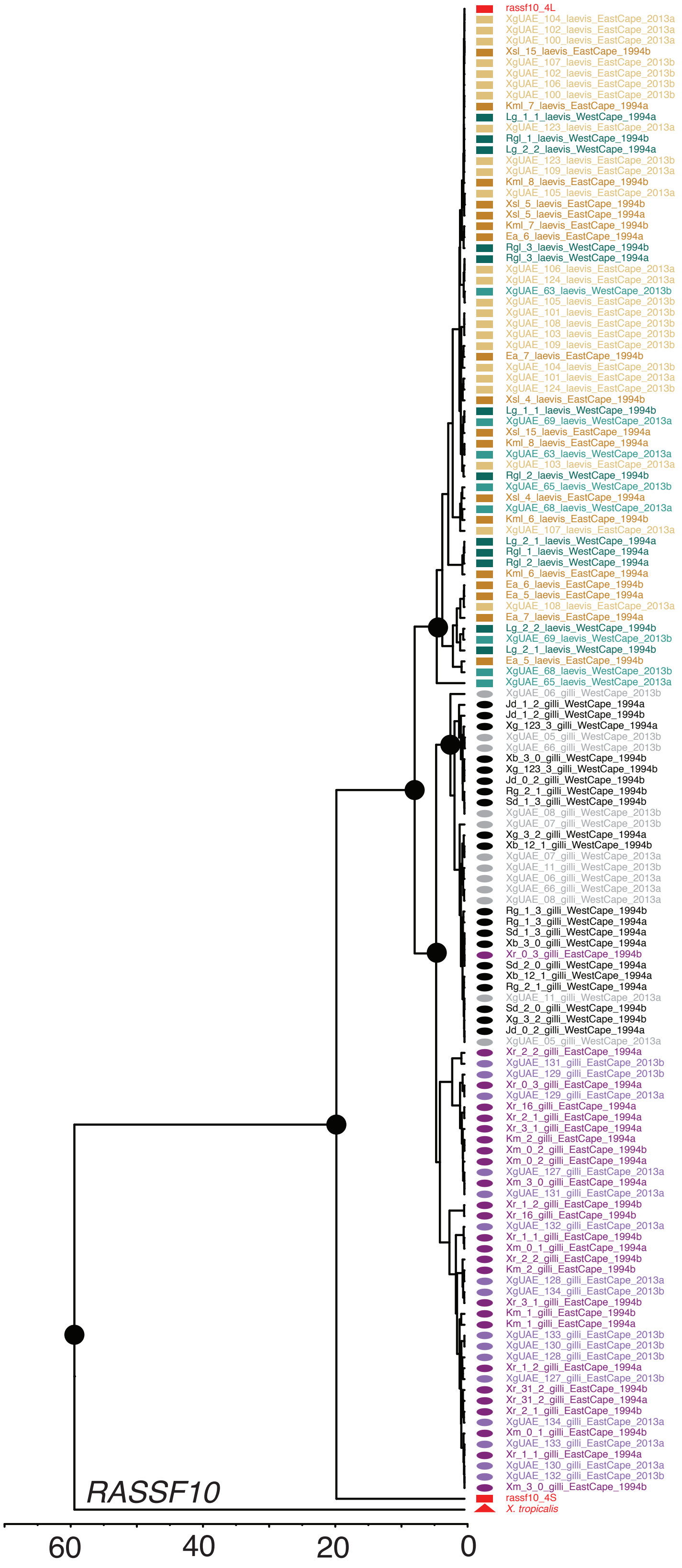

Figure S1. continued.

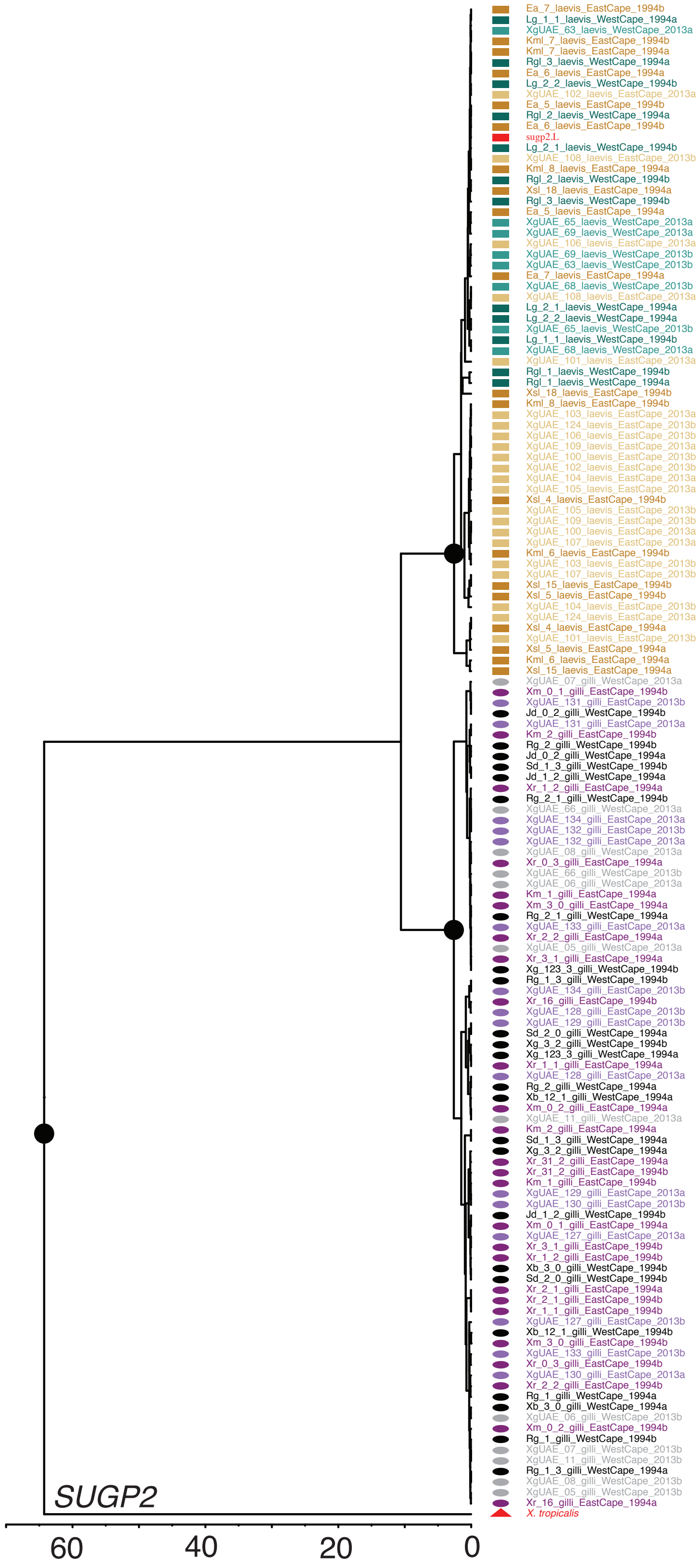

Figure S1. continued.

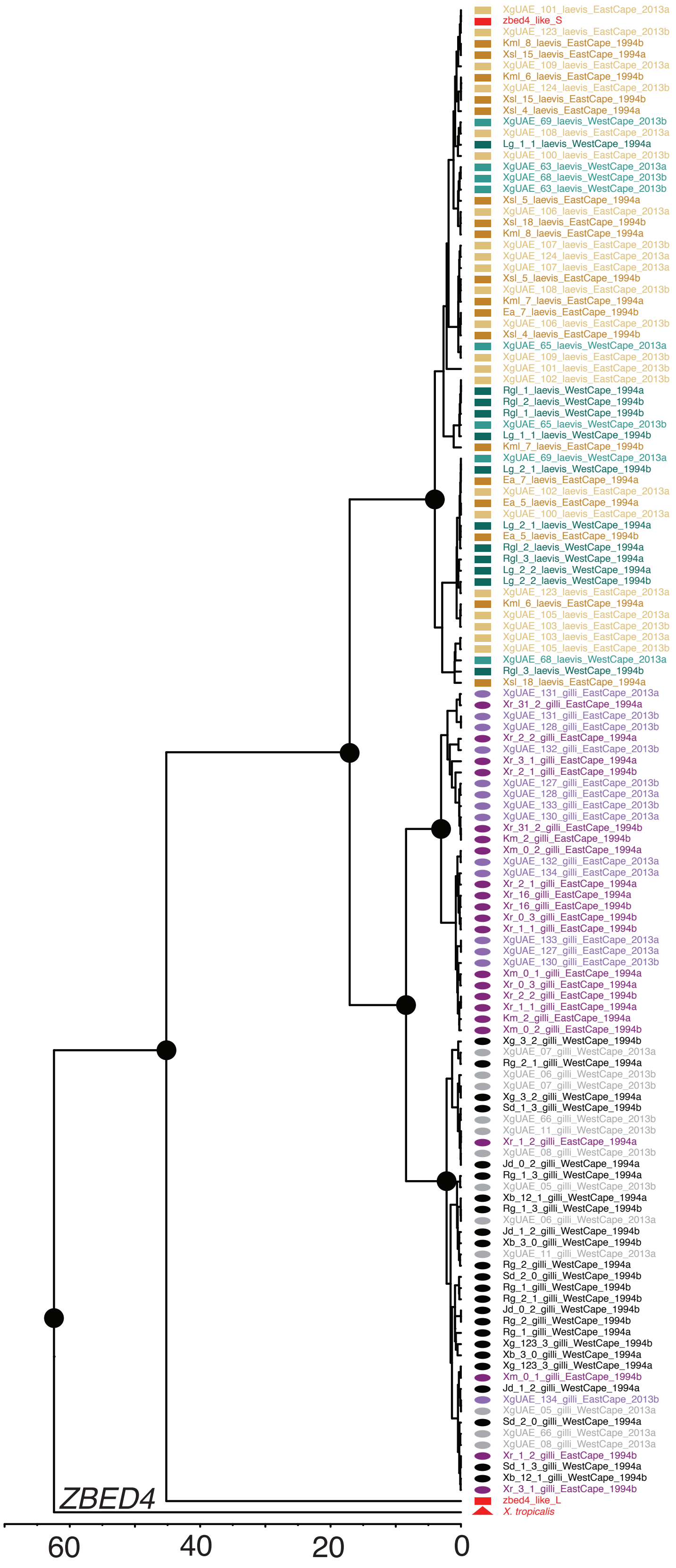

Figure S1. continued.

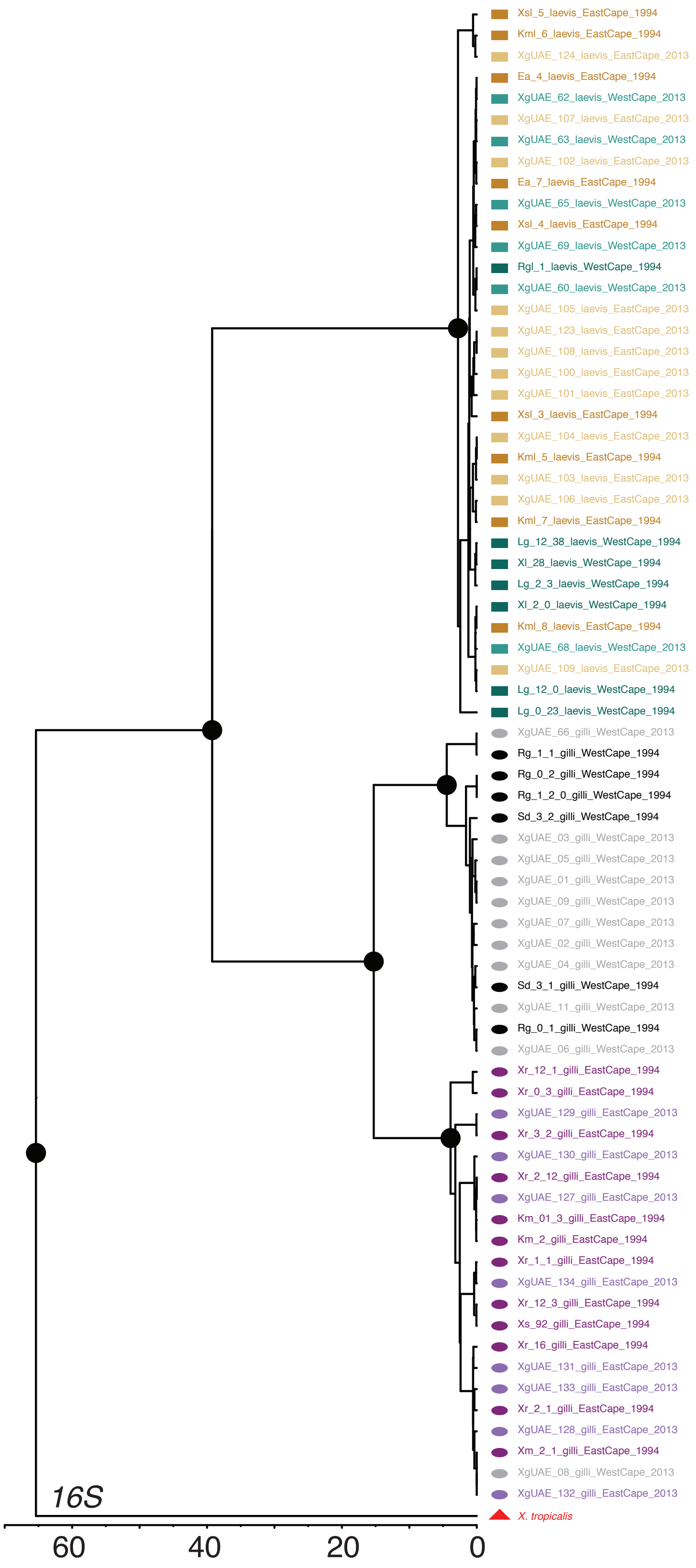

Figure S1. continued.

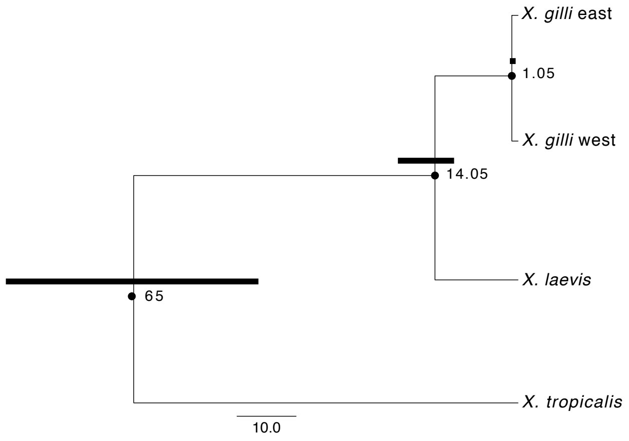

Figure S2. *BEAST analysis of the same 10 loci used in the Structure analysis (see Materials and Methods). The root of the tree was scaled to a 65 my divergence time from *X. tropicalis* [63]. Error bars represent 95% HDP on the height of a node.
